# Innovative pH-responsive alginate-coated rosuvastatin-loaded chitosan nanoparticles: a targeted approach to inhibiting HMGB1-activated RAGE/TLR4-NFκB signaling in colonic inflammation in rats

**DOI:** 10.3389/fphar.2025.1546010

**Published:** 2025-06-18

**Authors:** Elsayed A. Elmorsy, Sameh Saber, Zubida M. Al-Majdoub, Rabab S. Hamad, Mustafa Ahmed Abdel-Reheim, Asmaa Ramadan, Norah Suliman Alsoqih, Mariam S. Alharbi, Hamad Alsaykhan, Alshaimaa A. Farrag, Hanan Eissa, Rasha Abdelhady, Abousree T. Ellethy, Mostafa M. Khodeir, Hossam A. Elsisi, Syed Suhail Ahmed, Ahmed Y. Kira

**Affiliations:** ^1^ Department of Pharmacology and Toxicology, College of Pharmacy, Qassim University, Buraidah, Saudi Arabia; ^2^ Department of Clinical Pharmacology, Faculty of Medicine, Mansoura University, Mansoura, Egypt; ^3^ Department of Pharmacology, Faculty of Pharmacy, Delta University for Science and Technology, Gamasa, Egypt; ^4^ Centre for Applied Pharmacokinetic Research, University of Manchester, Manchester, United Kingdom; ^5^ Biological Sciences Department, College of Science, King Faisal University, Al Ahsa, Saudi Arabia; ^6^ Department of Pharmacology, College of Pharmacy, Shaqra University, Shaqra, Saudi Arabia; ^7^ Department of Biochemistry, Faculty of Pharmacy, Delta University for Science and Technology, Gamasa, Egypt; ^8^ Department of Pediatrics, College of Medicine, Qassim University, Buraidah, Saudi Arabia; ^9^ Department of Medicine, College of Medicine, Qassim University, Buraidah, Saudi Arabia; ^10^ Department of Anatomy and Histology, College of Medicine, Qassim University, Buraidah, Saudi Arabia; ^11^ Department of Anatomy, College of Medicine, University of Bisha, Bisha, Saudi Arabia; ^12^ Department of Pharmacology, College of Medicine, University of Bisha, Bisha, Saudi Arabia; ^13^ Pharmacology and Toxicology Department, Faculty of Pharmacy, Fayoum University, Fayoum, Egypt; ^14^ Pharmacology and Toxicology Department, Faculty of Pharmacy, Egypt ian Chinese University, Cairo, Egypt; ^15^ Department of Basic Oral Sciences and Dental Education, Biochemistry Division, College of Dentistry, Qassim University, Buraidah, Saudi Arabia; ^16^ Department of Pathology, College of Medicine, Qassim University, Buraidah, Saudi Arabia; ^17^ Department of Pathology, Faculty of Medicine, Cairo University, Cairo, Egypt; ^18^ Department of Clinical Pharmacology, Faculty of Medicine, Zagazig University, Zagazig, Egypt; ^19^ Department of Microbiology and Immunology, College of Medicine, Qassim University, Buraidah, Saudi Arabia; ^20^ Department of Pharmaceutics, Faculty of Pharmacy, Delta University for Science and Technology, Gamasa, Egypt

**Keywords:** rosuvastatin/pH-responsive nanoparticles, inflammation, colonic delivery system/colitis, sodium alginate/chitosan nanoparticles, HMGB1-RAGE/TLR4-NFκB, DSS/Sprague-Dawley rat colitis model

## Abstract

This study developed and optimized an innovative oral pH-dependent drug delivery system utilizing rosuvastatin-loaded chitosan nanoparticles (RSV-CSNPs) coated with sodium alginate (ALG). The goal was to protect RSV-CSNPs from degradation in the acidic gastric environment and facilitate targeted sustained release in the colon to address inflammatory bowel disease. Nanoparticles were initially prepared by ionic gelation. A subsequent ALG coating process was optimized using a 2^3^ factorial design. The optimal ALG-coated formulation demonstrated minimal drug loss (0.88% ± 0.09%), desirable particle size (407.2 ± 1.95 nm), and suitable zeta potential (−27.13 ± 1.36 mV). *In vitro* release tests highlighted the superiority of ALG-coated RSV-CSNPs, with significantly reduced RSV release in simulated gastric fluid (6.8% ± 1.06% after 2 h) compared to uncoated nanoparticles (38.45% ± 1.79%), affirming the protective effectiveness of the ALG coating. Extended-release studies at colonic pH (6.8) demonstrated sustained RSV release suitable for colon-specific targeting. *In vivo* assessments in a dextran sodium sulfate (DSS)-induced rat model of colitis revealed that ALG-RSV-CSNPs significantly outperformed both plain RSV and RSV-CSNPs. Treatment notably decreased colonic inflammation, disease severity scores, macroscopic damage, and oxidative stress indicators. Additionally, histopathological analyses showed remarkable restoration of colon tissue integrity, crypt preservation, and mucosal protection in animals treated with ALG-RSV-CSNPs. Mechanistically, ALG-RSV-CSNPs effectively attenuated colitis by significantly inhibiting the HMGB1-triggered RAGE/TLR4-NFκB inflammatory signaling pathway. Treatment resulted in substantial reductions in key inflammatory markers, including HMGB1, RAGE, TLR4 expression, NFκB activity, pro-inflammatory cytokines (TNF-α and IL-6), and apoptosis marker caspase-3. The anti-inflammatory actions were further supported by reduced neutrophil infiltration, lipid peroxidation, and enhanced antioxidant enzyme activities (SOD and GSH levels). The study identified HMGB1, RAGE, TLR4, and NFκB as critical biomarkers predicting disease activity. Correlation analysis highlighted strong positive associations among these markers, underscoring their collective involvement in colitis pathogenesis and emphasizing the multitarget therapeutic efficacy of ALG-RSV-CSNPs. Overall, this study demonstrates that the optimized pH-responsive ALG-coated RSV-CSNPs significantly enhance the therapeutic outcomes of RSV in colonic inflammation through targeted delivery and sustained release. These nanoparticles represent a promising strategy for effectively managing ulcerative colitis and related inflammatory bowel diseases. Future clinical studies are warranted to validate these findings and facilitate translation into human therapeutic applications.

## Graphical Abstract

**Figure:**
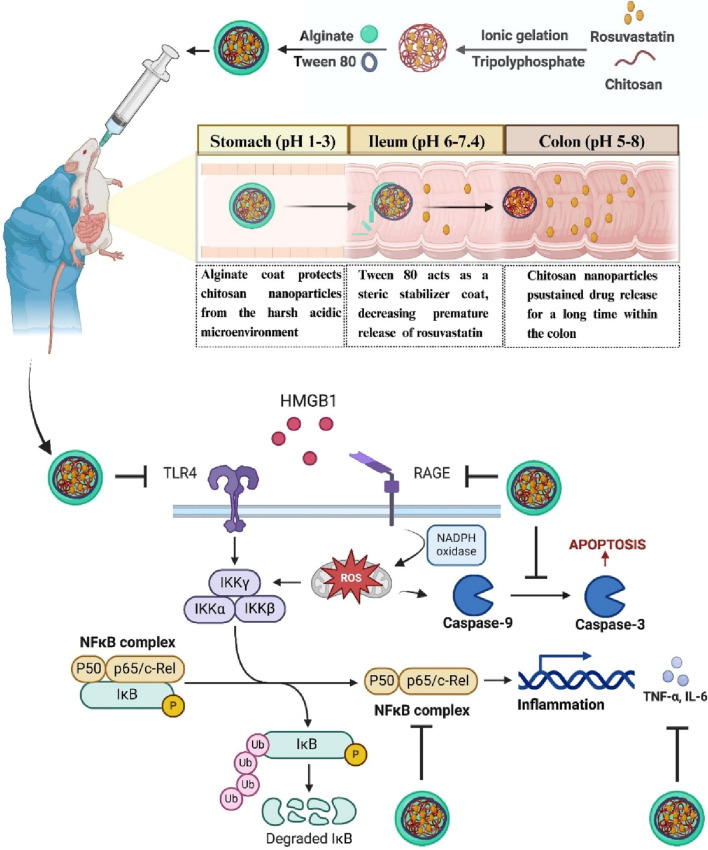
Visual abstract.

## 1 Introduction

Inflammatory bowel diseases such as ulcerative colitis (UC) have become a major global health challenge, with increasing numbers of cases (Buie et al., 2023). UC is characterized by chronic mucosal inflammation, which can progress to colorectal cancer over time (Guo and Wang, 2023; Zhang et al., 2023). This condition is often devastating, and until now, there has been no real cure, underscoring the need for novel therapeutic options for UC patients.

Cholesterol-lowering statins have shown promise in treating UC (Vital et al., 2023; Gou et al., 2024). Results indicate that statins may reduce the need for anti-inflammatory drugs and colectomy rates (Bai et al., 2021). Rosuvastatin (RSV), a potent 3-hydroxy-3-methylglutaryl coenzyme A (HMG-CoA) reductase inhibitor, has been shown to have anti-inflammatory effects beyond its known lipid-lowering properties, including inhibition of nuclear transcription factor kappa-B (NFκB) (Burma et al., 2014), tumor necrosis factor alpha (TNF-α) (Chen et al., 2023), IL-6 (Gualtero et al., 2017), and caspase-3 (Famer and Crisby, 2004), and reduction of oxidative stress in animal models (Ren et al., 2021; Saadat and Boskabady, 2021).

The colon poses significant physiological challenges for oral administration (Gaur et al., 2019). Being located in the last part of the gastrointestinal tract (GIT), the colon necessitates drug passage through different GIT segments, and each segment represents its challenges, including varied enzyme density, transit times, and pH values (Hasan et al., 2025). The stomach exhibits an acidic environment (pH 1–3). In contrast, the small intestine shifts to a relatively neutral environment (pH 6–7.4). Finally, the colonic environment demonstrates further variation (pH 5–8). As a result, an advanced delivery system is required to overcome these challenges and effectively target the colon.

Nanotechnology-based materials, particularly natural polymers, can overcome the GIT physiological challenges. These systems shield drugs from the harsh acidic and enzymatic environment of GIT and exploit physiological differences along GIT to achieve site-specific drug release (Sunoqrot et al., 2017). Among natural polymers, chitosan (CS) and ALG are the most studied because they are biocompatible, biodegradable, and can form stable nanoparticles (NPs) under mild conditions (Rostami, 2022; Kira et al., 2024a). CS is renowned for its mucoadhesive properties, which enable it to adhere to the GIT mucosal surfaces, thereby enhancing penetration and extending drug retention time (Chen S. et al., 2022). One major problem is that CS is soluble under acidic conditions, thereby impeding oral administration, which could lead to premature drug release in the stomach, resulting in fewer drugs being available within the colon (Einbu et al., 2007). However, this limitation can be overcome by using ALG as coating material. The stability of ALG under acid conditions makes it act as a protective layer, preventing CS dissolution in the stomach and ensuring drug delivery into the colon (Zhang et al., 2022). Additionally, under mild conditions, anionic ALG can readily interact with cationic chitosan nanoparticles (CSNPs) through electrostatic forces, without the need for organic solvents.

The toll-like receptor 4 (TLR4) and the receptor for advanced glycation endproducts (RAGE) function as the main high mobility group box 1 (HMGB1) receptors (Yang et al., 2020). It has been acknowledged that excessive levels of extracellular HMGB1 have been linked to tissue damage (Andersson et al., 2018) and illnesses including inflammatory bowel diseases (IBDs) (Chandrashekaran et al., 2017; He et al., 2018). Moreover, NFκB and other distinct signaling molecules are activated by HMGB1. As a result of this signaling cascade, pro-inflammatory cytokines are produced which are essential to the etiology of chronic inflammatory diseases (Lotze and Tracey, 2005). Research also revealed that the interaction between HMGB1 and RAGE can influence TLR4 signaling, which may contribute to the inflammatory reactions seen in IBDs (Kokkola et al., 2005; Valdés-Ferrer et al., 2013). Furthermore, research has demonstrated that HMGB1 triggers the release of inflammatory cytokines through both RAGE and TLR4 (Valdés-Ferrer et al., 2013). These findings support HMGB1 as a promising therapeutic target for sterile inflammatory diseases for which there is presently no effective treatment.

This study aimed to optimize the ALG coating process of CSNPs to enhance the colonic delivery and thereby the anti-inflammatory effect of RSV for the treatment of colitis *via* oral administration. Five different formulations of rosuvastatin-loaded chitosan nanoparticles (RSV-CSNPs) were prepared through the ionic gelation method and *in vitro* characterized regarding particle size (PS), entrapment efficiency (EE%), and zeta potential (ZP). Moreover, the ALG coating process of the selected RSV-CSNPs was optimized using 2^3^ full factorial design, aiming to minimize drug loss (DL)% and premature release in the stomach or small intestine environment. The optimized coated formula (Fr2) was subjected to comprehensive *in vitro* and *in vivo* evaluations. *In vitro*, release study, morphological characteristics, and structural analysis *via* infrared spectroscopy were conducted. *In vivo*, adult male Sprague-Dawley rats were used to assess the therapeutic effectiveness of RSV, RSV-CSNPs, and alginate-coated rosuvastatin-loaded chitosan nanoparticles (ALG-RSV-CSNPs) on UC induced by dextran sodium sulfate (DSS). We investigated the possible anti-inflammatory function of the new formula ALG-RSV-CSNPs, with a particular focus on the RAGE/TLR4-NFκB signaling in the injured colon that is driven by HMGB1. It was expected that the optimized formulation could protect CSNPs from the acidic gastric environment *via* a pH-dependent release mechanism, making CSNPs available in a small intestine environment (pH 7.4) where CSNPs can decrease the premature release of RSV, increase drug concentration within the colon, and enhance RSV efficacy against colitis, offering an effective strategy for overcoming the challenges of colon-specific drug delivery.

## 2 Material and methods

### 2.1 Materials

Acros Organics Company (Geel, Belgium) provided chitosan (CS, CAS No. 9012-76) polymer with an average molecular weight of 100,000–300,000 Da and a deacetylation degree of 80%–85%. Sigma-Aldrich (St. Louis, MO, United States) supplied rosuvastatin (RSV, CAS No.147098-20-2), Na-alginate (ALG, CAS No. 9005-38-3), sodium tripolyphosphate (TPP, CAS No. 7758-29-4), ethanol (HPLC grade, CAS No. 64-17-5), acetonitrile (HPLC grade, CAS No. 75-05-8), and a dialysis cellulose membrane (MW cutoff 12,000 175 g/mol). Thermo Fisher Scientific (London, United Kingdom) supplied glacial acetic acid (CAS No. 64-19-7) and 0.22 μm plastic syringe filters. El-Nasr Pharmaceutical Chemicals Company (Al-Qalyubiyah, Egypt) supplied potassium dihydrogen phosphate and di-sodium hydrogen phosphate. Each chemical used was analytical grade and used as obtained. DSS was purchased from Sigma-Aldrich (Mw = ∼40,000).

### 2.2 Methods

#### 2.2.1 Preparation of RSV- loaded CSNPs

CSNPs were prepared, utilizing the ionic gelation method *via* crosslinking with Na-TPP (Nasr et al., 2023a). CS was dissolved in a 1% w/v acetic acid solution at a concentration range of 0.1%–0.3% w/v using a magnetic stirrer at room temperature and 600 rpm for 15 min. Na-TPP was dissolved in deionized water at a concentration of 0.1% w/v. A volume of 10 mL of Na-TPP solution was added slowly (2 mL/min) into 20 mL of chitosan solution, which was kept under constant stirring for 1 h at 600 rpm to ease crosslinking with Na-TPP. The formed nanodispersion was sonicated for 10 min using a bath sonicator. The same methodology was conducted for the development of RSV-CSNPs, with the addition of an aliquot of 0.5 mL of DMSO solution equivalent to 20 mg RSV. The optimization of RSV-CSNPs involved the preparation of five distinct formulations, each with a weight ratio of CS to Na-TPP ranging from 2:1 to 6:1. Subsequently, the optimal formulation was chosen for the ALG coating process.

#### 2.2.2 Design and preparation of ALG-coated RSV-CSNPs

##### 2.2.2.1 Design of the coating process

Using Minitab 19 software, a three-factor, two-level (2^3^) full factorial design was developed for the optimization of the ALG coating process (Table 1). The selected dependent factors were ALG concentration (3% and 4% w/v), stirring time (10 and 20 min), and the presence or absence of Tween 80. The effect of these variables was optimized with a goal to achieve the possible minimum values of the following responses: PS, DL%, release% at simulated gastric fluid after 2 h (Q_2_%), and release% at simulated intestinal fluid after 4 h (Q_6_%).

**TABLE 1:** Coded units of the selected factors and their corresponding targeted responses as developed in the 2^3^ factorial design

Factors	levels		Responses	Target
A = ALG concentration (%)	3	4	R1 = PS	Minimize
B = Stirring time (min)	10	20	R2 = DL%	Minimize
C = Tween 80 concentration (%)	-	1	R3 = Q_2_%	Minimize
Coded values	Low (−1)	High (+1)	R4 = Q_6_%	Minimize

ALG, Na-alginate; R, response; PS, particle size, DL%, drug loss percentage; Q_2_%, drug release percentage after 2 h at simulated gastric fluid; Q_6_%, drug release percentage after 4 h at simulated intestinal fluid.

##### 2.2.2.2 Preparation of ALG-coated RSV-CSNPs

The coating process involved the electrostatic interaction between positively charged CSNPs and negatively charged ALG (Li et al., 2021). For the preparation of ALG-coated CSNPs, the optimal RSV-CSNPs and blank NPs were lyophilized and dispersed in phosphate buffer (pH 5.5) at a concentration of 1% w/v with the absence or presence of 1% w/v tween 80. ALG was dissolved in deionized water at a concentration of three or 4% w/v. The CSNPs dispersion was added slowly into the ALG solution under mild stirring at room temperature for 10 or 20 min. The mixture was centrifuged at 3,000 rpm for 10 min, and the suspended particles (ALG-coated CSNPs) were separated and dispersed in an aqueous solution of CaCl_2_ (0.5 mM) (Li et al., 2008), kept under stirring for an additional 10 min for the cross-linking of ALG layers on the CSNPs’ surface. Finally, the coated particles were centrifuged (3,000 rpm for 10 min), separated, washed with deionized water, and freeze-dried overnight. Eight different formulations of ALG-coated CSNPs were prepared according to Table 2.

**TABLE 2:** The composition of the prepared RSV-loaded ALG-coated CS NPs according to the developed 2^3^ factorial design.

Formula	Coded levels			ALG (% w/v)	Stirring time (min)	Tween 80 (% w/v)
	A	B	C			
Fr1	−1	−1	−1	3	10	−
Fr2	−1	−1	+1	3	10	1
Fr3	−1	+1	−1	3	20	−
Fr4	−1	+1	+1	3	20	1
Fr5	+1	−1	−1	4	10	−
Fr6	+1	-1	+1	4	10	1
Fr7	+1	+1	−1	4	20	−
Fr8	+1	+1	+1	4	20	1

#### 2.2.3 Characterization of RSV-CSNPs and ALG-coated CSNPs

##### 2.2.3.1 Size distribution and surface charge

The PS, PDI, and the surface charge of the prepared CSNPS and ALG-coated CSNPS were evaluated using Zetasizer (Malvern, United Kingdom) equipped with Malvern software version 7.01. Samples were diluted with deionized water in a ratio of 1:10. All measurements were conducted three times at room temperature.

##### 2.2.3.2 Determination of EE and DL%

For CSNPs, the EE% and loading efficiency (LE%) were assessed by the calculation of the initial total RSV amount (*A*_*0*_) and the free non-entrapped RSV amount (*A*_*1*_). *A*_*0*_ was determined by mixing 1 mL of the nanodispersion with 0.5 mL of glacial acetic acid to dissolve the polymeric coat and get the total amount. *A*_*1*_ was determined by the centrifugation of 1 mL of nanodispersion for 1 h at 3,000 rpm, followed by the separation of the supernatant. The EE and LE% were calculated according to Equations 1 and 2, respectively.

$$EE\;\left(\%\right)=\frac{A_{0}-A_{1}}{A_{0}}\times 100$$ (1)

$$LE\;\left(\%\right)=\frac{A_{0}-A_{1}}{Total\;NPs\;weight}\times 100$$ (2)

The DL% within the ALG coating process was determined by analyzing the free drug amount (*W*_*1*_) within the supernatant after centrifugation of ALG-coated CSNPs just before the crosslinking process with CaCL_2_ solution. The DL% was calculated according to Equation 3, using the previously determined entrapped amount (*A*_*0*_ - *A*_*1*_) of CSNPs as the initial total amount (*W*_*0*_).

$$DL\;\left(\%\right)=\frac{W_{1}}{W_{0}}\times 100$$ (3)

RSV concentration analysis was conducted using a spectrofluorometric method at an emission wavelength of 547 nm after excitation at 276 nm. The calibration curve was linear at a concentration range of 0.2–20 μg/mL with a high correlation coefficient of 0.9993.

##### 2.2.3.3 *In vitro* release study

The cumulative release percentage of RSV from the optimized CSNPs, different ALG-coated CSNPS formulations, and plain drug (all equivalent to 1 mg RSV) was assessed using the dialysis bag method. A volume of 1 mL of different formulations was enclosed in a dialysis bag (cellulose membrane-MWCO 12,000–14,000 Da) and immersed in a 40 mL release medium with different pH values simulating GIT conditions: dilute HCL solution of pH 1.2 related to stomach pH, phosphate buffered saline (PBS) of pH 7.4 related to small intestine pH, and PBS of pH 6.8 related to colon pH. The first 2 h of the experiment were conducted at pH 1.2, then the dialysis bags were removed and immersed in pH 7.4 for an additional 4 h, and finally immersed in pH 6.8 for 48 h. At different time intervals, a volume of 2 mL of the release medium was withdrawn for RSV analysis and replaced with the same volume of the corresponding fresh medium. The amount of RSV released was determined using the established spectrofluorometric method at an emission wavelength of 547 nm. The same methodology was conducted for plain NPs to be used as a blank during RSV analysis to exclude any interference.

#### 2.2.4 Optimization of ALG-coated RSV-CSNPs

The response optimization method was utilized in accordance with the developed factorial experimental design to ascertain the optimal circumstances for choosing the most optimized formulation. This calculation was performed using Minitab 19 software. A specific objective was established for the optimization approach, which necessitated achieving the minimum attainable values for PS, DL%, Q2%, and Q6% (Table 1). The chosen optimized formulation was then subjected to additional *in vitro* evaluation regarding morphological and structural characteristics, as well as comprehensive *in vivo* evaluation in rats’ model of colitis.

#### 2.2.5 Morphological and structural characteristics of the optimized formula

The morphological characteristics of the optimized CSNPs and ALG-coated CSNPs were evaluated using a transmission electron microscope (TEM) (JEOL, model 1200, Japan). After dilution of the optimized formulations with deionized water, one drop was added to the grid and left for drying. Then, a drop of uranyl acetate was added to the dried sample before being examined.

The structural characteristics of RSV, CS, ALG, uncoated CSNPs, and ALG-coated CSNPS were analyzed by observing their corresponding characteristic peaks using a Fourier transform infrared (FTIR) spectrophotometer (Shimadzu, model 8300, Japan). Approximately 5 mg of samples were grinded with potassium bromide at a constant ratio, and their corresponding peaks were detected at a frequency range of 4,000 to 400 cm^−1^.

#### 2.2.6 Animal study

Adult male Sprague-Dawley rats weighing 250 ± 20 g were purchased from the Faculty of Pharmacy, Delta University for Science and Technology (FPDU) and used in this study. During the acclimation or experimental periods, rats were housed in polycarbonate cages, with stainless steel wire lids and wood chip bedding. Each cage contained four rats. The room was maintained at 22°C ± 2°C with 55% ± 10% relative humidity and a 12-h light/dark cycle (lights on at 07:00 a.m.). Rats had *ad libitum* access to a standard pellet diet and tap water. During all procedures, handling was kept minimal, and efforts were made to minimize pain and distress, and the cages were routinely sanitized and cleaned. A preliminary experiment was conducted to collect pilot data and used to perform a power analysis in G*Power 3.1.9.7, which determined the number of animals required to achieve statistical significance while limiting animal use. All procedures followed the ARRIVE guidelines and complied with EU Directive 2010/63 on the protection of animals used for scientific purposes. The Research Ethics Committee at FPDU, Egypt granted ethical approval under the approval number FPDU24120/6. At the end of the study, rats were euthanized using an overdose of sodium pentobarbital (200 mg/kg, intraperitoneally (Pang and Laferriere, 2020)). Death was confirmed by the absence of a heartbeat and respiration.

##### 2.2.6.1 Experimental design

As described in Table 3, fifty six rats were divided into seven groups (n = 8 for each), as follows: The CTRL group did not receive any treatment and served as the baseline control; The ALG-RSV-CSNPs group received treatment with rosuvastatin-loaded chitosan nanoparticles coated with Na-alginate for 14 days; The DSS/ALG-CSNPs, rats were treated with 3% DSS (w/v) for 7 days to induce colitis and halted for the next 7 days (Kishimoto et al., 2000) and received alginate-coated chitosan nanoparticles as an intervention for 14 days; The DSS group received only DSS. This group served as the untreated disease control group; The DSS/RSV group was treated with DSS to induce colitis, followed by treatment with plain resveratrol (10 mg/kg/day. PO) for 14 days; The DSS/RSV-CSNPs group was treated with DSS and received resveratrol-loaded chitosan nanoparticles for 14 days. Lastly, the DSS/ALG-RSV-CSNPs group was induced with DSS and treated with alginate-coated resveratrol-loaded chitosan nanoparticles for 14 days. The residual water from DSS ingestion was observed to make sure that there were no drug-dependent differences in the amount of drinking water consumed by drug-treated DSS rats and their corresponding DSS control group.

**TABLE 3:** Experimental design.

Group	Treatment during days (1–7)	Treatment during days (8–14)
CTRL	Healthy control; no treatment	
ALG-RSV-CSNPs	Healthy rats treated with alginate-coated RSV-CSNPs	
DSS/ALG-CSNPs	3% DSS + alginate-coated CSNPs	alginate-coated CSNPs
DSS	3% DSS in tap water	—
DSS/RSV	3% DSS + Resveratrol (10 mg/kg/day, PO)	Resveratrol (10 mg/kg/day, PO)
DSS/RSV-CSNPs	3% DSS + Resveratrol (10 mg/kg/day, PO)	RSV-loaded chitosan nanoparticles
DSS/ALG-RSV-CSNPs	3% DSS + alginate-coated RSV-CSNPs	alginate-coated RSV-CSNPs

CTRL, Control; DSS, Dextran sulfate sodium; RSV, Rosuvastatin; CSNPs, Chitosan nanoparticles; ALG, Sodium alginate; ALG-CSNPs, Alginate-coated chitosan nanoparticles; RSV-CSNPs, Rosuvastatin-loaded chitosan nanoparticles; ALG-RSV-CSNPs, Alginate-coated rosuvastatin-loaded chitosan nanoparticles; PO, Per os (oral administration).

#### 2.2.7 Rationale of rosuvastatin selection

Rosuvastatin is a statin with documented anti-inflammatory effects beyond cholesterol lowering, including inhibition of NFκB (Burma et al., 2014), TNF-α (Chen et al., 2023), IL-6 (Gualtero et al., 2017), and caspase-3 (Famer and Crisby, 2004), and reduction of oxidative stress in animal models (Ren et al., 2021; Saadat and Boskabady, 2021). Additionally, our preliminary work showed that rosuvastatin mitigated DSS-induced colitis in rats. Its FDA approval and well-characterized safety profile support its repurposing for UC. Furthermore, since patients with IBD often present with low HDL and elevated LDL levels (Sappati Biyyani et al., 2010), rosuvastatin may offer dual benefits by addressing both systemic inflammation and lipid abnormalities, providing a rationale for exploring its use as a targeted therapy in colitis.

##### 2.2.7.1 Rationale of rosuvastatin dosage

It has been reported that oral rosuvastatin at 1 mg/kg/day demonstrated a significant reduction in the myocardial levels of HMGB1 and RAGE in adriamycin-challenged rats after 6 weeks (Zhang et al., 2018). Additionally, oral rosuvastatin at 2 mg/kg/day administrated for 2 weeks revealed improved azithromycin-evoked biochemical and oxidative stress marker alterations (Mansour et al., 2021). However, our preliminary studies and previously published work (Saber et al., 2021a) revealed that the lowest effective oral dose of rosuvastatin in mitigating acute colitis induced by DSS was 10 mg/kg/day for a duration of 14 days.

#### 2.2.8 Microscopical examination

Colon samples fixed in 10% neutral buffered formalin were processed and embedded in paraffin. Sections of 5 µm thickness were cut from the blocks and mounted on glass slides. These sections were deparaffinized in xylene and rehydrated through a graded ethanol series to distilled water. For hematoxylin and eosin (H&E) staining, sections were stained with Harris’s hematoxylin for 5 min, rinsed in running tap water, and differentiated in 1% acid alcohol. After bluing in alkaline water, they were counterstained with eosin for 2 min. The stained sections were then dehydrated in graded ethanol, cleared in xylene, and mounted with coverslips for microscopic examination. Five sections were analyzed per rat, with six rats included in each group. In each section, 10 randomly selected, non-overlapping fields were examined. Edema, crypt architecture, and inflammation were evaluated using an Olympus CX23 microscope equipped with a digital camera. A blinded pathologist performed the assessment using the scoring criteria described in Table 4.

**TABLE 4:** Scoring criteria for edema, crypt atrophy, and inflammation.

Score	Edema	Crypt atrophy	Inflammation or ulceration features
0	Absence of edema	Absence of crypt atrophy	Lack of inflammation
1	Minimal edema	Minimal crypt atrophy	There is just mucosal layer-specific inflammation or ulceration
2	Mild edema	Mild crypt atrophy	Ulceration or inflammation affects the mucosa and submucosa layers
3	Moderate edema	Moderate crypt atrophy	The muscularis propria layer is involved in ulceration or inflammation
4	Severe edema	Severe crypt atrophy	The colon's whole wall, including the serosa, is inflamed, and there are isolated ulcerations
5	—	—	Extensive inflammation and ulceration affecting the serosa and all layers of the colon wall
6	—	—	Intestinal wall perforation resulting in ulceration and inflammation throughout all layers

Immunohistochemical labeling of NFκB p65 was performed to liver specimens cut into a 5 µm-thickness. This process comprised fixation, dewaxing, dehydration, citrate buffer heat-mediated antigen retrieval and finally incubation with rabbit p65 (eBioscience, CA, United States; diluted to 1:1,000) polyclonal primary antibodies overnight at 4°C followed by an HRP-conjugated goat secondary antibody. Percentage of NFκB immunopositive cells (counted and expressed as a percentage of total cells) was assessed in randomly selected 10 different non-overlapping fields. Five sections were analyzed per rat, with six rats included in each group.

#### 2.2.9 Image analysis

A representative region of interest (ROI) from each group was selected to contain at least three crypts within the region. This ensured that the analysis focused on regions representative of mucosal inflammation and crypt architecture. Then, the images were processed using segmentation techniques. Crypts were isolated based on their morphology using thresholding with ImageJ (version 1.54f, NIH, United States). The crypts were differentiated from the surrounding tissues. Crypts were examined to assess the degree of preservation or damage. In addition, contour plots were generated using MATLAB (R2023a) with a colored gradient scale to display regions of varying intensity to provide a visual representation of tissue structural changes. All image analyses were performed in a blinded manner.

#### 2.2.10 Assessment of colonic weight-to-length ratio and macroscopic damage index (MDI)

The colonic weight-to-length ratio was measured as another tissue inflammation and edema indicator. After euthanasia (12.5 mg/kg Xylazine and 87.5 mg/kg Ketamine), the entire colon was excised, cleaned, weighed, and measured. Additionally, a blinded pathologist evaluated macroscopic damage (Saber et al., 2023) following the criteria described in Table 5.

**TABLE 5:** Criteria for the assessment of macroscopic damage.

Score	Macroscopic Feature
0	Not damaged
1	Hyperemia with no ulcers
2	Ulcer that is linear with non-significant inflammation
3	Ulcer that is linear with inflammation at a single location
4	Two or more locations with inflammation/ulceration
5	Two or more major locations of inflammation/ulceration, or one location measuring 1 cm or more in length of inflammation/ulceration
6–10	Damage extending more than two centimeters down the colon, with a point increase of one for each centimeter involved

#### 2.2.11 Assessment of colonic oxidative stress biomarkers

Myeloperoxidase (MPO) activity, an indicator of neutrophil infiltration, was measured using a colorimetric assay by following the protocol from Abcam, Cambridge, United Kingdom (Cat. #: ab105136). Malondialdehyde (MDA), a marker of lipid peroxidation, was measured using a colorimetric assay (Bio-diagnostic, Egypt; Cat. #: MD2529). Superoxide dismutase (SOD) activity, an antioxidant enzyme, was measured using a colorimetric assay kit (Bio-diagnostic; Cat. #: SD2521). Additionally, reduced glutathione (GSH), a major intracellular antioxidant, was determined using a colorimetric assay (Bio-diagnostic; Cat. #: GR2511) as per the manufacturer’s instructions. All measurements were conducted in duplicate.

#### 2.2.12 ELISA-based quantitative determination of colonic HMGB1, RAGE, TLR4, NFκB, TNF-α, IL-6, and active caspase-3

HMGB1 and RAGE levels were measured using sandwich ELISA kits from LifeSpan BioSciences Inc. (Seattle, WA, United States; Cat. #: LS-F4039 for HMGB1 and LS-F23591 for RAGE). TLR4 expression was assessed using a sandwich ELISA kit (Cloud-Clone Corp., Houston, TX, United States; Cat. #: SEA753Ra). NFκB activity was determined using a DNA-binding ELISA-like assay kit (Abcam, United Kingdom; Cat. #: ab210613) with nuclear extracts prepared using a cytoplasmic and nuclear extraction kit (Abcam; Cat. #: ab113474), and activity was measured in arbitrary units based on absorbance at 450 nm. TNF-α and IL-6 levels were quantified using sandwich ELISA kits (R&D Systems, Minneapolis, MN, United States; Cat. #: RTA00 for TNF-α and R6000B for IL-6). Lastly, active caspase-3 levels were measured using a colorimetric enzyme activity assay kit from Abcam, United Kingdom; Cat. #: ab39401. All measurements were conducted in duplicate following the manufacturer’s instructions.

#### 2.2.13 Correlation analysis

Three analysis approaches were used to examine the relationships between RAGE, TLR4, and NFκB activity in the context of rosuvastatin intervention. Multivariate scatterplot matrix included histograms depicting the distribution of individual variables and scatterplots with linear regression lines to provide insight into the pairwise relationships between these variables. Additionally, a correlogram was constructed based on the Pearson correlation coefficients to quantify the strength of the linear relationships between the three variables to provide numerical support for the visual interpretations. Furthermore, a gradient-boosting partial dependence plot (PDP) was employed to analyze the nonlinear effects of RAGE and TLR4 on NFκB activity. The PDP provided a detailed view of the marginal influence of RAGE and TLR4 on NFκB activity by isolating the effects of each variable while keeping other variables constant. All figures and analyses were performed using Python 3.10 in a JupyterLab environment. The multivariate scatterplot matrix with regression lines and histograms was created using Seaborn 0.12, while Matplotlib 3.6 handled plot rendering. The correlogram was built from Pearson correlation coefficients calculated with Pandas 1.5 and visualized using Seaborn. Gradient boosting models were trained with Scikit-learn 1.2 to analyze the nonlinear influence of RAGE and TLR4 on NFκB activity. Partial dependence plots were generated using PartialDependenceDisplay from the same library. NumPy 1.23 supported underlying data operations throughout.

#### 2.2.14 Assessment of disease activity index (DAI)

The DAI was determined by a blinded gastroenterologist through assessing three parameters as described in Table 6 (Alshahrani et al., 2025). The individual scores are summed to give the overall DAI. Higher DAI scores indicate increased colitis severity.

**TABLE 6:** Criteria for evaluating colitis severity (disease activity).

Parameter	Score	Features
Diarrhea	0	for normal stool
	1	for soft stool
	2	for very soft stool
	3	for watery diarrhea
Weight loss	0	for no loss
	1	for 1–5% loss
	2	for 6–10% loss
	3	for 11–15% loss
	4	for 16–20% loss
Bloody stool	0	for no hemoccult
	1	for hemoccult-positive
	2	for traces of blood
	3	for severe rectal bleeding

#### 2.2.15 Feature importance analysis in predicting disease activity using random forest regression

To determine the importance of each parameter in predicting DAI (disease severity), a random forest regressor model was used as a hypothesis generator The forest regressor model is a machine-learning model used for regression tasks. The dataset was organized into a pandas DataFrame 1.5, where the parameters served as independent features (X) and DAI as the dependent variable (y). This model was used to predict DAI and determine feature importance, given its reliable performance in calculating the significance of features based on their contribution to reducing prediction error. The model was configured with default estimators and a random state of 42 for reproducibility, and it was implemented using the scikit-learn 1.2 library. Feature importance scores were extracted after training to evaluate the contribution of each parameter to predicting DAI. A lollipop chart (Matplotlib 3.6) was used to visualize the feature importance scores.

#### 2.2.16 Assessment of the effects of different parameters on colitis severity (displayed as DAI) based on correlation coefficients

To evaluate the importance of each parameter in relation to the disease activity, a lollipop chart was constructed using Matplotlib based on the correlation coefficients. Each stem represents a parameter, and the dot indicates its value.

#### 2.2.17 Statistical analysis

Minitab 21 was used for factorial design statistical analysis using multiple regression analysis. The other findings were statistically examined with GraphPad Prism 9 (California, United States). The t-test assessed the statistical difference between the two data sets. The results from different groups were compared using the one-way ANOVA test and the Tukey’s multiple comparison confirmation test in the case of parametric measurements. Normality of the data was assessed using the Shapiro-Wilk test. For the inflammation, edema, crypt atrophy scores, the Kruskal–Wallis test followed by Dunn’s as a *post hoc* test was utilized. Six experimental replicates per group were analyzed and all biochemical assays were performed in duplicate (technical replicates) for each biological sample. Every possible comparison between the study groups was considered and the data are presented as the mean ± standard deviation (parametric data) or as the median ± IQR (nonparametric data), with a p-value < 0.05 indicating significant differences between groups.

## 3 Results

### 3.1 RSV analysis

**FIGURE 1:**
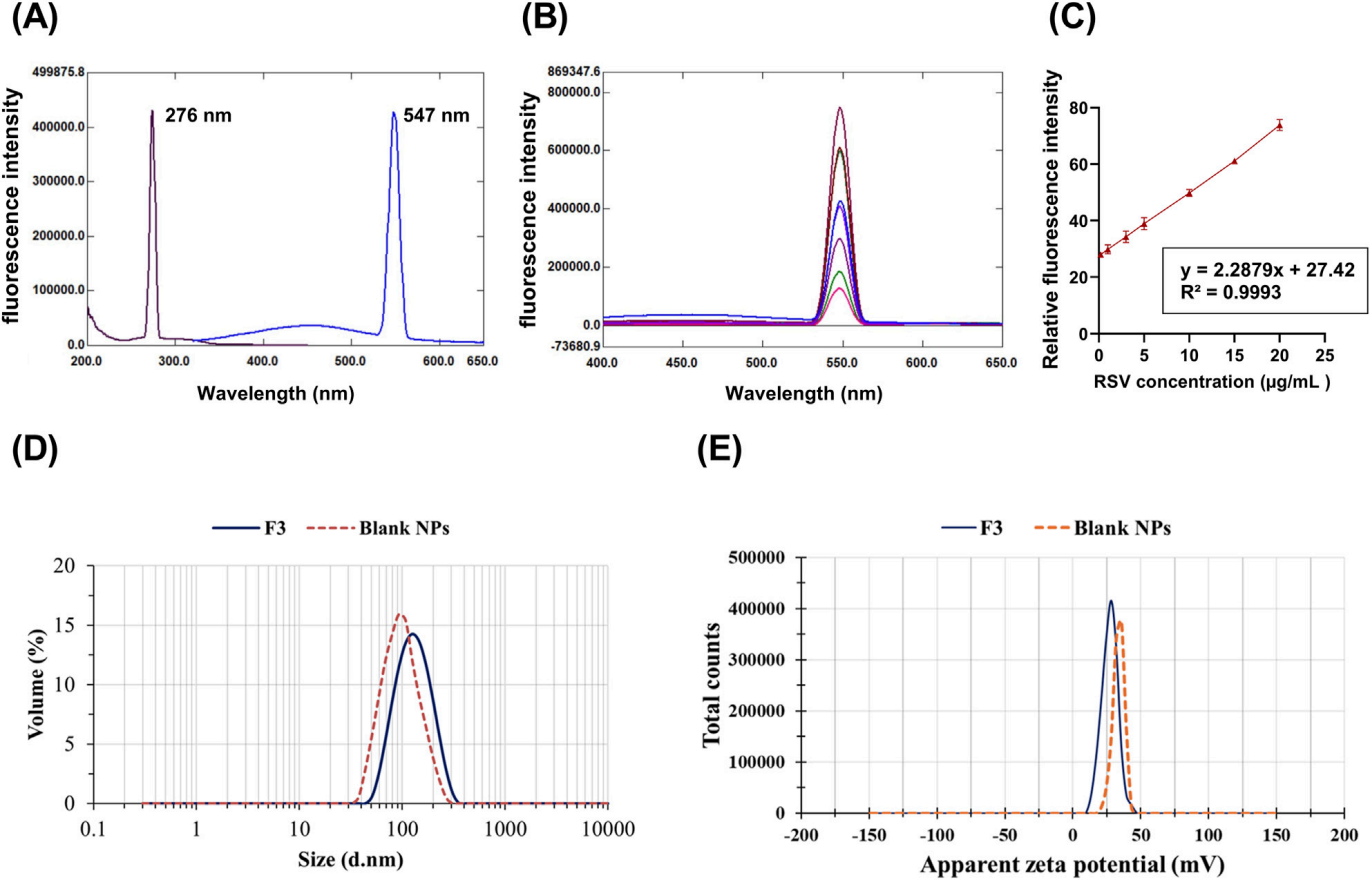
Fluorescence intensity for RSV at an excitation wavelength of 276 nm and an emission of 547 nm **(A)**. The calibration curve for RSV determination, RSV concentration (µg/mL) against mean relative fluorescence intensity (± SD, n = 5) **(B)**. Emission spectra of different concentrations of RSV at the predetermined emission wavelength **(C)**. The mean particle size **(D)** and apparent zeta potential **(E)** of the optimized formulation (F3) and blank NPs.

The spectrofluorometric method was selected for the determination of RSV as it is notable for its eco-friendly, rapidity, and sensitivity (Andrade-Eiroa et al., 2013). After using different solvents, ethanol demonstrated the maximum fluorescence intensity with an emission wavelength of 547 nm after excitation at 276 nm (Figure 1A). Good linearity and results were obtained as demonstrated by the successive increase in fluorescence intensity (Figure 1B). The calibration curve was linear over RSV concentration range 0.2–20 μg/mL with a high correlation coefficient of 0.9993 (Figure 1C).

### 3.2 Characterization of RSV-CSNPs

Table 7 summarizes the *in vitro* characterization results of the prepared RSV-CSNPs. The PS of the prepared formulations was in the range of (139.1–244.8 nm). There was a gradual decrease in the PS upon increasing the CS/Na-TPP mass ratio till reaching 4:1, which may be due to the comparatively elevated Na-TPP levels at lower mass ratios. Higher levels of TPP facilitate the development of inter- and intramolecular cross-links, resulting in the generation of larger particles (Kira et al., 2024b). However, the significant increase in PS at a CS/Na-TPP mass ratio above 4:1 may result from the higher amount of chitosan employed in these ratios, therefore augmenting the number of polymer particles that constitute each vesicle (Elmorsy et al., 2024). The PDI value of RSV-CSNPs ranged from 0.312 to 0.517, indicating a relatively uniform distribution, particularly in formulations F2 and F3.

**TABLE 7:** Summary of the *in vitro* characterization results of the prepared RSV-CSNPs.

Formula	CS: TPP mass ratio	PS (nm)	ZP (mV)	EE (%)	LE (%)	PDI
F1	2:1	178.3 ± 1.76	+18.27 ± 0.81	86.36 ± 0.28	28.54 ± 0.001	0.399 ± 0.01
F2	3:1	147.4 ± 3.58	+23.52 ± 0.95	83.43 ± 0.66	26.34 ± 0.002	0.343 ± 0.06
F3	4:1	139.1 ± 1.01	+28.9 ± 1.18	89.72 ± 1.15	23.14 ± 0.002	0.312 ± 0.01
F4	5:1	210.4 ± 1.16	+30.3 ± 1.04	82.51 ± 1.79	20.62 ± 0.005	0.488 ± 0.04
F5	6:1	244.8 ± 4.25	+33.8 ± 0.96	75.78 ± 1.36	17.74 ± 0.003	0.517 ± 0.02

The ZP of RSV-CSNPs was between +18.27 ± 0.81 and 33.8 ± 0.96 mV, and it had a positive charge because of the CS’s positive amino groups (+NH_3_). Higher CS/Na-TPP mass ratios correlate with an increase in CS concentration and its positively charged amino groups, leading to an increase in the ZP value. Additionally, the LE% and EE% of RSV-CSNPs were in the range of (17.74%–28.54%) and (75.78%–89.72%), respectively. There was no notable difference between the EE% of the prepared formulations, except for F5, which demonstrated a significant decrease in the EE% (75.78% ± 1.36%). This reduction in the EE% may be related to the high concentration of CS, which increases the gelation medium viscosity, making it more challenging for the drug to move and encapsulate within the NP core (Delan et al., 2020).

Formulation F3 was chosen for the ALG-coating process, as it demonstrated the smallest PS (139.1 ± 1.01 nm) and PDI (0.312 ± 0.01). In addition, it exhibited the highest EE% (89.72% ± 1.15%), with a relatively high ZP of (+28.9 ± 1.18 mV). These parameters collectively make F3 optimal for the ALG coating process. Figures 1D,E show the mean diameter and ZP of the optimal formulation (F3) and blank NPs, respectively. Compared to blank NPs, there was a significant increase in PS and a significant decrease in ZP for F3. The observed increase in PS could result from the entrapped drug molecules, increasing the PS. The negatively charged carboxylate of RSV, which is loaded on the NPs surface, could be the cause of the subsequent decrease in the positive surface charge value.

### 3.3 Design and characterization of ALG-coated RSV-CSNPs

According to the established factorial design, eight different formulations (Fr1–Fr8) of ALG-coated NPs were prepared and characterized for PS, DL%, drug release % (Q_2_% and Q_6_%), and ZP (Table 8).

**TABLE 8:** *In vitro* characterization results of ALG-coated RSV-CSNPs.

Formula	PS (nm)	EE%	LE%	DL (%)	Q_2_ (%)	Q_6_ (%)	ZP (mV)
Fr1	382.07 ± 2.87	84.51 ± 1.25	18.52 ± 0.38	4.53 ± 0.36	12.91 ± 1.77	36.21 ± 1.68	−27.86 ± 0.51
Fr2	407.2 ± 1.95	88.22 ± 0.89	22.26 ± 0.05	0.88 ± 0.09	6.812 ± 1.06	25.41 ± 2.03	−27.13 ± 1.36
Fr3	411.4 ± 1.31	83.67 ± 0.46	17.68 ± 0.17	5.46 ± 0.37	9.56 ± 1.31	35.67 ± 0.63	−29.03 ± 1.07
Fr4	447.7 ± 2.28	87.12 ± 1.27	21.02 ± 0.39	2.21 ± 0.31	5.75 ± 1.38	25.71 ± 2.07	−25.92 ± 0.68
Fr5	472.11 ± 2.84	80.83 ± 0.75	14.84 ± 0.21	8.39 ± 0.43	7.69 ± 0.53	36.49 ± 1.64	−32.8 ± 1.67
Fr6	527.08 ± 2.76	84.02 ± 1.13	18.83 ± 0.19	4.42 ± 0.44	4.87 ± 1.08	24.33 ± 0.82	−30.76 ± 1.58
Fr7	498.69 ± 1.76	75.16 ± 1.02	9.76 ± 1.27	13.16 ± 1.06	4.32 ± 0.49	33.88 ± 1.21	−36.43 ± 1.05
Fr8	593.63 ± 5.17	82.9 ± 1.41	16.91 ± 0.75	6.78 ± 0.97	4.02 ± 0.06	25.93 ± 1.02	−33.56 ± 2.07

PS, particle size; EE%, entrapment efficiency; LE%, loading efficiency; DL%, drug loss percentage; Q2%, drug release percentage after 2 h at simulated gastric fluid; Q6%, drug release percentage after 4 h at simulated intestinal fluid; ZP, zeta potential; PDI, polydispersity index.

The ZP of ALG-coated formulations ranged from −25.92 ± 0.68 (Fr4) to −36.43 ± 1.05 mV (Fr7). The negative values are correlated with the anionic nature of ALG, which coats CSNPs, imparting a net negative surface charge. The PS ranged from 382.07 ± 2.87 (Fr1) to 593.63 ± 5.17 nm (Fr8). The presence of ALG coating around the CSNPs resulted in an increase in the size of the particles in comparison to the uncoated formulation (Fr3), which demonstrated a significantly smaller PS (139.1 ± 1.01 nm). This increase in size is attributed to the formation of a thicker alginate layer around the CSNPs.

**FIGURE 2:**
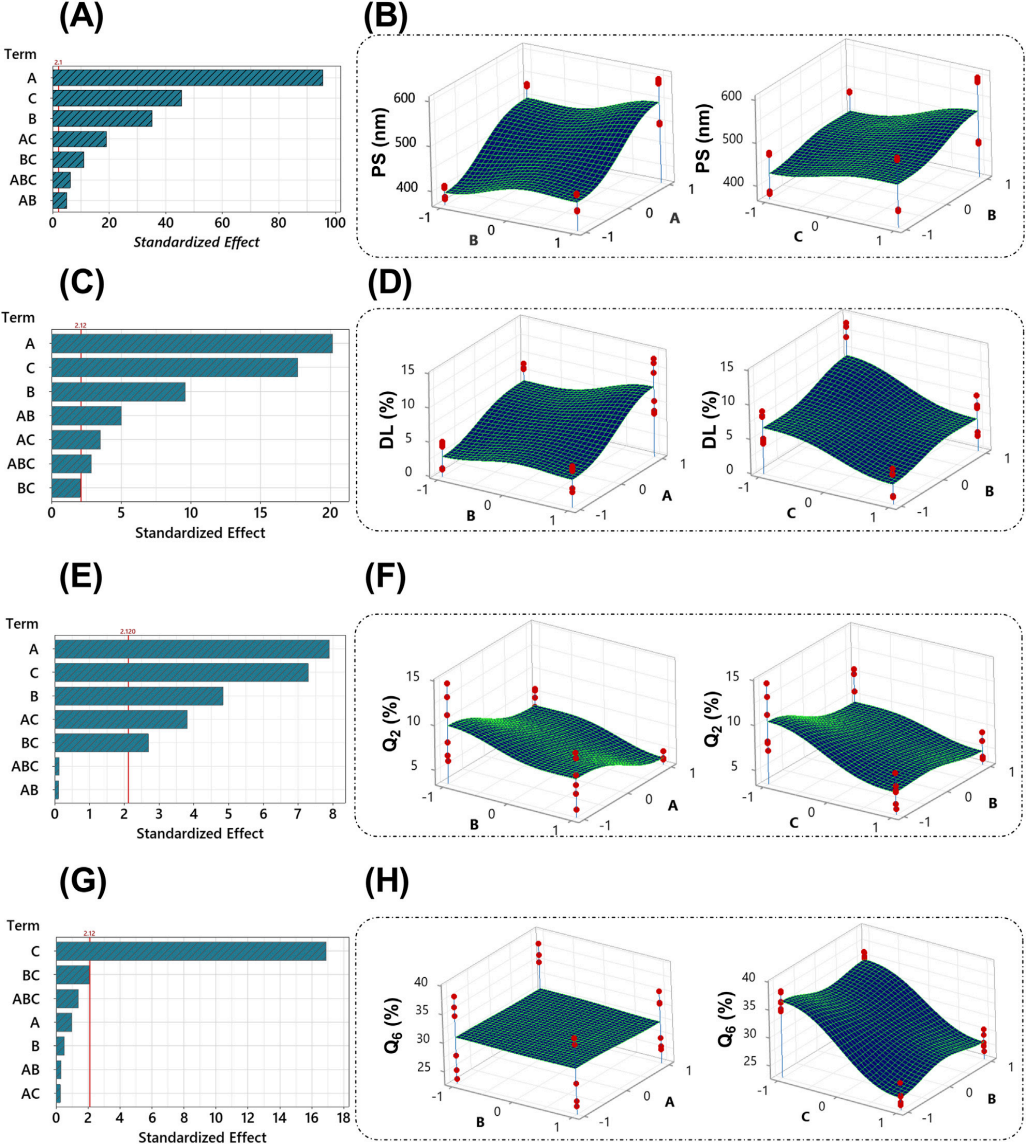
Pareto graphs depict the standardized impacts on PS **(A)**, DL% **(C)**, Q_2_% **(E)**, and Q_6_% **(G)**. The 3D surface plots of PS **(B)**, DL% **(D)**, Q_2_% **(F)**, and Q_6_% **(H)** with variances of ALG concentration, stirring time, and tween 80 concentration. The red line on the Pareto chart has been assigned as a reference for statistical significance. The given significance level is denoted by α = 0.05. The red dots on the 3D surface plots represent the values of different runs at the selected levels for each factor.

According to the Pareto chart that describes the effect of the selected factors on PS (Figure 2A), all factors significantly (*p* < 0.05) affect PS, factor A (ALG concentration) being the most significant factor, followed by B (stirring time) and C (Tween 80). Figure 2B demonstrates the direct relationship between these factors and PS. The following regression equation describes this effect (Equation 4):

$$PS\;\left(nm\right)=467.487+55.393\;A+20.370\;B+26.418\;C+2.912\;AB+11.060\;AC+6.393\;BC+3.600\;ABC$$ (4)

Regarding DL%, the coated formulations showed varied values ranging from 0.88 ± 0.09 (Fr2) to 13.16% ± 1.06% (Fr7). The Pareto chart (Figure 2C) revealed that all factors significantly (*p* < 0.05) affect DL% except the interaction BC. The most significant factor was the concentration of ALG, followed by the concentration of tween 80 and the stirring time. The stirring time and ALG concentration directly affected the DL%, whereas the presence of tween 80 significantly lowered it (Figure 2D). The regression (Equation 5) further corroborates these findings.

$$DL\;\left(\%\right)=5.732+2.462\;A+1.172\;B-2.158\;C+0.611\;AB-0.429\;AC-0.251\;BC-0.349\;ABC$$ (5)

The RSV release % after 2 h at pH 1.2 (Q2%) ranged from 4.02 ± 0.06 (Fr8) to 12.91 % ± 1.77% (Fr1). The variations in Q2% were influenced significantly (*p* < 0.05) by all three factors, except for the interactions between factors A and B, and the combined interactions (ABC), as shown in the Pareto chart (Figure 2E). The 3D surface plots reveal an inverse relationship between these factors and Q_2_% (Figure 2F), suggesting that higher concentrations of alginate, extended stirring time, and the presence of Tween 80 decrease the initial drug release in gastric conditions. The following regression equation describes this effect (Equation 6):

$$Q2\;\left(\%\right)=6.996-1.764\;A-1.080\;B-1.628\;C+0.024\;AB+0.851\;AC+0.601\;BC+0.029\;ABC$$ (6)

**FIGURE 3:**
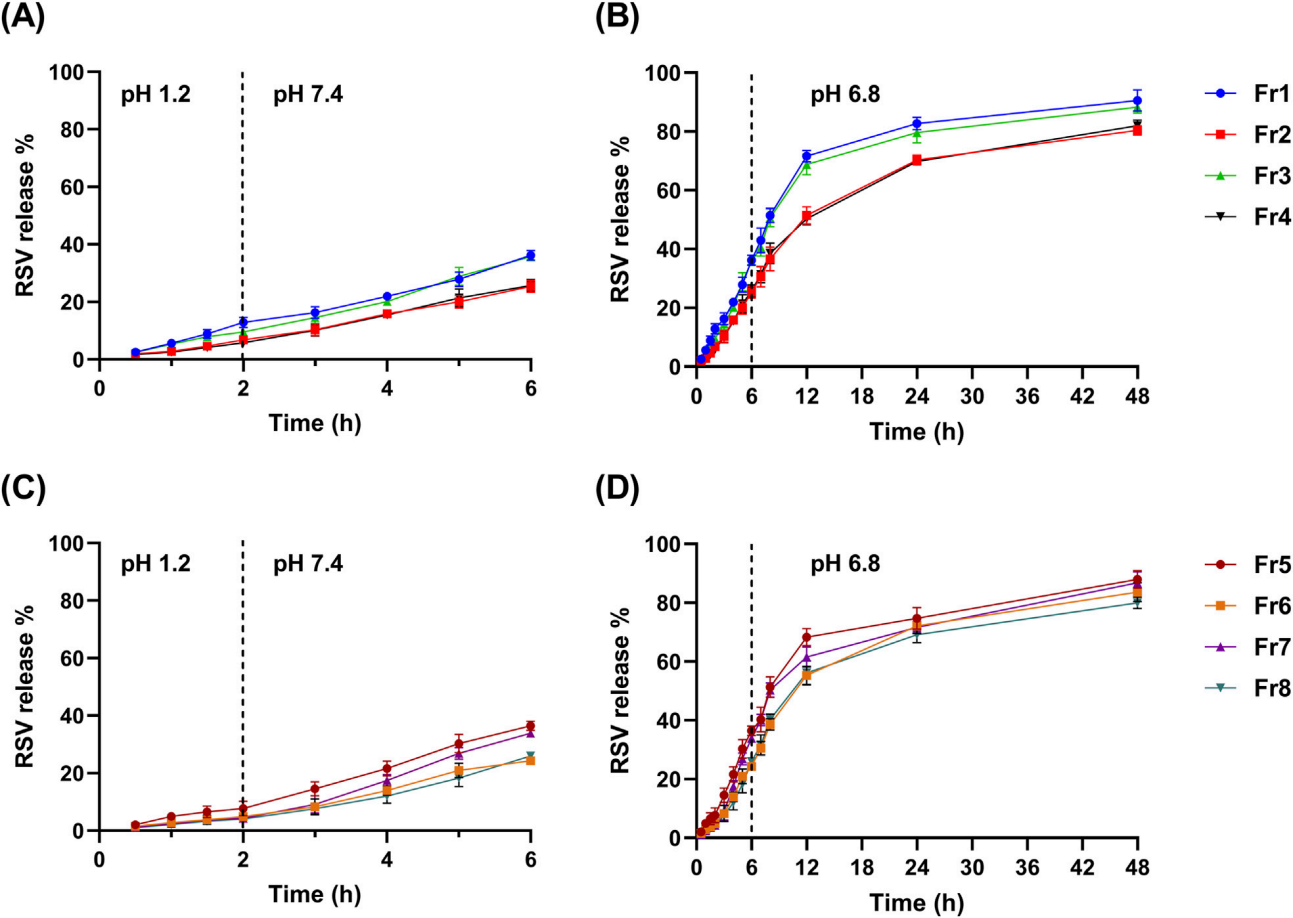
The mean release % of RSV (± SD, n = 3) from the ALG-coated CSNPS formulations over time at various pH conditions simulating different parts of the gastrointestinal tract. **(A, B)** show the release profiles of formulations Fr1, Fr2, Fr3, and Fr4: **(A)** 2 h at pH 1.2 and 4 h at pH 7.4, **(B)** continued release at pH 6.8 for 48 h. Panels C and D present the release profiles of formulations Fr5, Fr6, Fr7, and Fr8: **(C)** 2 h at pH 1.2 and 4 h at pH 7.4, **(D)** continued release at pH 6.8 over 48 h.

After 4 h in simulated intestinal fluid (pH 7.4), Q6% ranged from 24.33 ± 0.82 (Fr6) to 36.49% ± 1.68% (Fr5). Unlike Q2%, the Q6% was mainly influenced by the presence of Tween 80 while the other factors demonstrated a non-significant effect (*p* > 0.05), as indicated by the Pareto chart (Figure 2G). The addition of Tween 80 significantly reduced the drug release in the intestinal environment (Figure 2H). The regression equation (Equation 7) further corroborates these findings. The release profiles of RSV from the coated formulations in different media to simulate the GIT environment are shown in Figure 3.

$$Q6\;\left(\%\right)=30.458-0.297\;A-0.157\;B-5.112\;C-0.096\;AB+0.081\;AC+0.630\;BC+0.422\;ABC$$ (7)

### 3.4 Optimization of ALG-coated CSNPs

**FIGURE 4:**
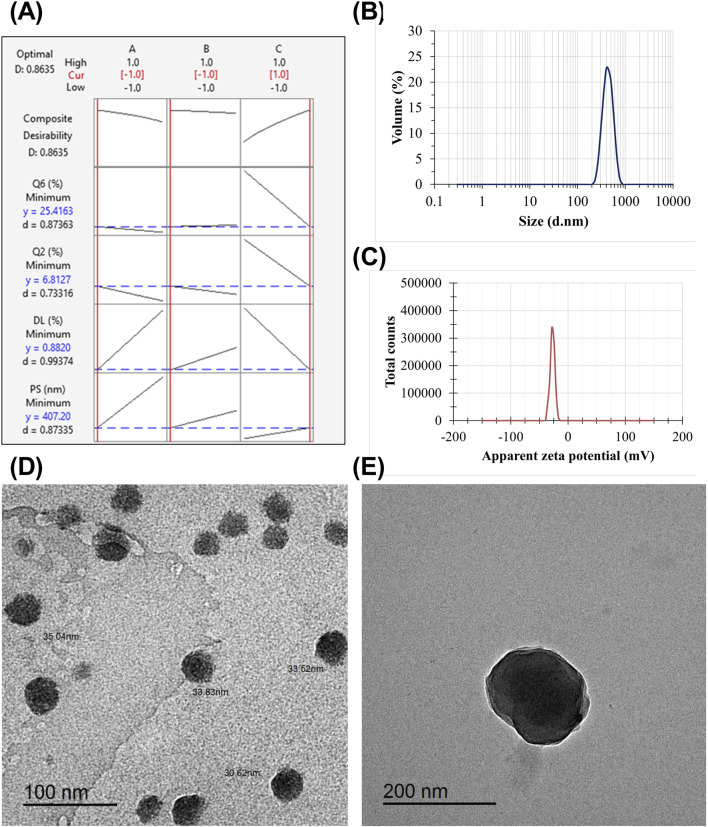
The response optimization plot visually represents the influence of each individual factor (columns) on the respective response or composite desirability (rows) **(A)**. The red numbers shown at the top of a column and the vertical red lines on the graph indicate the present factor settings of the selected optimal formulation. The vertical red line on the left corresponds to the low level (−1), as in the case of ALG concentration and stirring time. In contrast, with the tween 80 factor, the red line is located on the right side. The responses of the optimized formulation are represented by horizontal blue lines and numerical values. “Cur” represents the current configurations of the factors, “D” represents the overall desirability, “y” represents the value of the response, and “d” gives the specific desirability for each response. The mean PS **(B)** and the mean ZP **(C)** of the optimized ALG-coated CSNPs. TEM micrographs of the optimized CSNPs **(D)** and ALG-coated CSNPs **(E)**.

Following the application of the response optimization method, the optimization plot (Figure 4A) demonstrates that the optimal formula consistently yielded the best results for all evaluated parameters, consisting of lower-level values for factors A (3% w/v ALG) and B (10 min stirring) and a higher-level value for factor C (1% w/v tween 80), which aligns with formulation (Fr2). The PS and ZP of Fr2 are shown in Figures 4B,C, respectively.

### 3.5 Characterization of the optimized ALG-coated formulation (Fr2)

#### 3.5.1 Morphological characteristics

The TEM micrographs show that the optimized formulation has a spherical shape depicting the ALG layer around CSNPs (Figure 4E). The TEM analysis revealed that all uncoated CSNPs had a small size range within the nanometer scale and appeared to be mostly spherical in shape. Furthermore, the absence of aggregation or structural irregularities highlighted the integrity of the vesicles (Figure 4D). Additionally, there was a significant increase in particle diameter of ALG-coated NPs compared to uncoated CSNPs. It is noteworthy that the PS obtained from Zetasizer is higher than that obtained from TEM micrographs. Previous studies have demonstrated similar observations (Nasr et al., 2023b; Nasr et al., 2024). The discrepancy between the hydrodynamic size obtained from Zetasizer and the dry size obtained from TEM is primarily due to the presence of hydration and the ALG coating around the CSNPs. The Zetasizer measures the hydrodynamic size, which includes the contribution of the water molecules surrounding the particles and the ALG coating, leading to an increase in the apparent size. In contrast, TEM provides the dry size by visualizing the solid core of the nanoparticles. The ALG coating adds an additional layer to the particle surface, which also contributes to the higher hydrodynamic size observed in the Zetasizer measurements. This is consistent with previous studies, which have shown that the hydrodynamic size is typically larger than the dry size due to the presence of hydration and surface coatings (Bagre et al., 2013; Alshawwa et al., 2023).

#### 3.5.2 *In vitro* release

**FIGURE 5:**
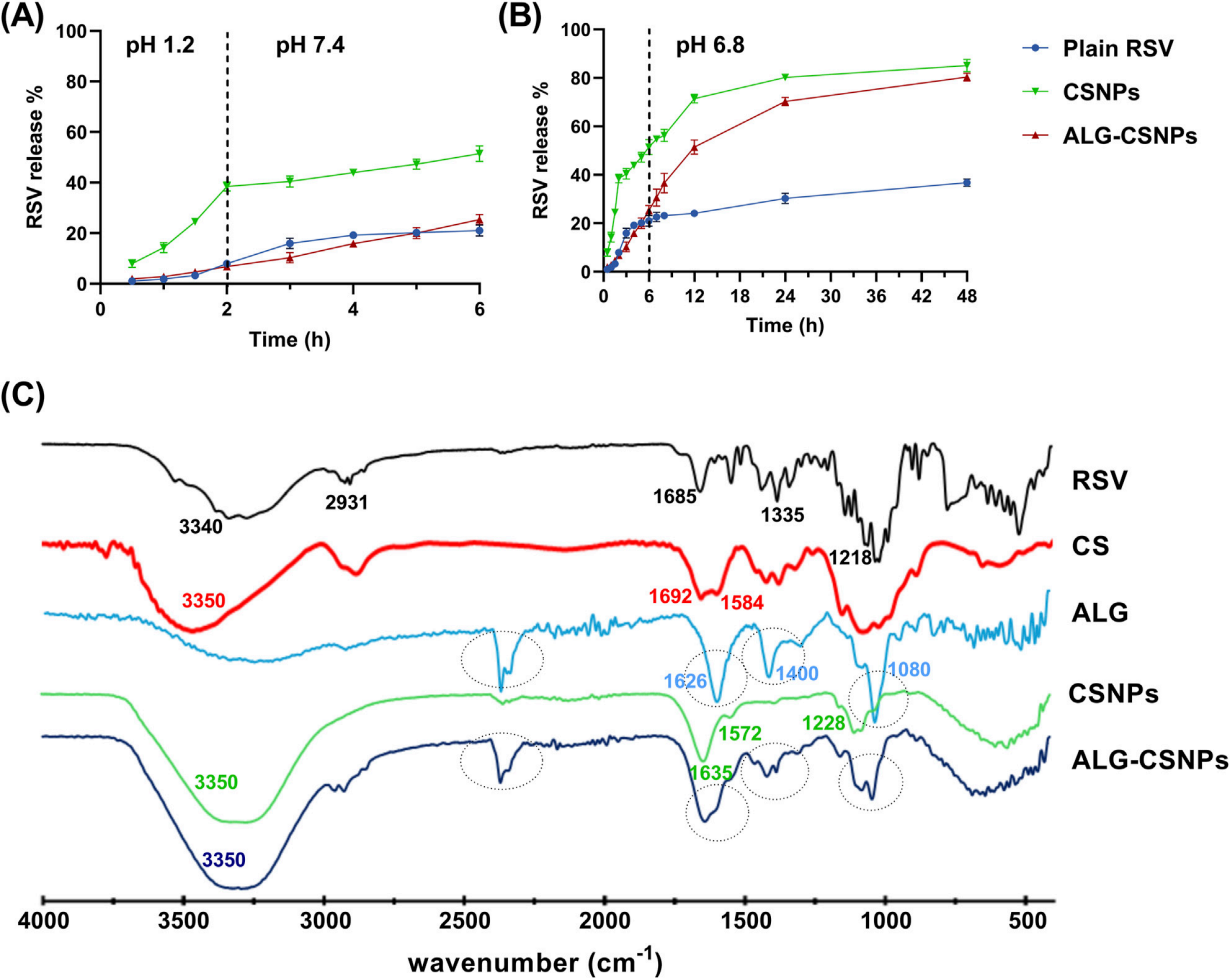
The mean release % of RSV (± SD, n = 3) from the plain RSV, uncoated CSNPs, and ALG-coated CSNPS formulations over 6 h at pH 1.2 and pH 7.4 **(A)**, and the extended-release profile at pH 6.8 for 48 h **(B)**. The FTIR of plain RSV, chitosan (CS), Na-alginate (ALG), uncoated CSNPs, and ALG-coated CSNPs **(C)**.

A comparative release study was conducted to assess the release pattern of plain RSV suspension, uncoated RSV-CSNPs (F3), and optimized ALG-coated RSV-CSNPs (Fr2). As shown in Figure 5A, the uncoated CSNPs demonstrated a significantly higher release percentage (38.45% ± 1.79%) compared to ALG-coated CSNPs (6.81% ± 1.06%) after 2 h at a stomach pH of 1.2. This trend continued at an intestinal pH of 7.4, where, after 4 h, the uncoated CSNPs demonstrated a significantly higher release (51.47% ± 3.12%) compared to ALG-coated CSNPs (25.41% ± 2.03%). Extending the release study to 48 h at pH 6.8 simulating colonic environments (Figure 5B), revealed a non-significant difference in RSV release % between uncoated CSNPs (85.12 ± 2.54) and ALG-coated CSNPs (80.39 ± 1.58). Regarding the plain RSV suspension, being BCS class II with poor solubility, it exhibited a generally limited drug release pattern in comparison to NPs formulations, highlighting the enhanced drug release characteristics of the NPs formulations.

#### 3.5.3 FTIR study

Figure 5C displays the FTIR spectra of RSV, CS, ALG, uncoated CSNPs (F3), and ALG-coated CSNPs (Fr2). The FTIR spectrum of RSV revealed characteristic peaks at 3,340 cm^−1^ (O–H stretching of carboxylic group), 2,931 cm^−1^ (N–H stretching), 1,685 cm^−1^ (C=O stretching of carboxylic group), 1,335 cm^−1^ (C–F stretching), and 1,218 cm^−1^ (S=O stretching) (Sarfraz et al., 2017). Notably, these characteristic peaks were absent in the CSNPs spectrum, suggesting the successful entrapment of RSV within the NPs core.

The spectrum of CS showed characteristic peaks around 3,350 cm^−1^ (O–H and N–H stretch overlapping), 1,692 cm^−1^ (C=O stretching of amide I), and 1,584 cm^−1^ (NH2 bending vibration). In the CSNPs spectrum, the peak of CS at 3,350 became broader, revealing the H-bond formation with Na-TPP. Additionally, the two peaks of CS at 1,692 cm^−1^ and 1,584 cm^−1^ shifted to 1,635 cm^−1^ and 1,572 cm^−1^, respectively. This observation aligns with previous reports, which attribute these shifts to crosslinking between the phosphoric groups of Na-TPP and the amino groups in CS (Agarwal et al., 2018; Amri et al., 2021). Moreover, a new peak at 1,228 cm^−1^ corresponding to P=O of Na-TPP was observed in the CSNPs spectrum, further supporting this crosslinking interaction.

Regarding ALG and ALG-coated CSNPs spectra, the ALG spectra exhibited characteristic peaks at 1,080 cm^−1^ (C-O-C stretching) related to its saccharide structure. Peaks at 1,626 and 1,400 cm^−1^ related to asymmetric and symmetric stretching peaks of carboxylate salt groups. These three distinct peaks were also identified in the FTIR spectrum of the ALG-coated CSNPs, distinguishing them from the uncoated CSNPs and confirming the presence of an ALG coating layer around the CSNPs. Moreover, the broad peak (3,350 cm^−1^) and amide bond peak (1,635 cm^−1^) of CSNPs became more intense in ALG-CSNPs spectra, confirming the electrostatic interaction between the negatively charged ALG and positively charged CSNPs.

### 3.6 Results of the *in vivo* rat study

#### 3.6.1 Mortality assessment

Mortality was recorded for each group as follows: no deaths occurred in the CTRL, ALG-RSV-CSNPs, DSS/RSV-CSNPs, or DSS/ALG-RSV-CSNPs rat groups; two deaths were observed in both the DSS/ALG-CSNPs group and the DSS group with a 25% mortality for each, and one death occurred in the DSS/RSV group with a 12.5% mortality. No signs of toxicity were observed in any group not exposed to DSS. Deaths observed in DSS-treated groups reflected the expected impact of colitis induction.

#### 3.6.2 Histological analysis of colonic crypt architecture as a measure of colonic tissue damage

**FIGURE 6:**
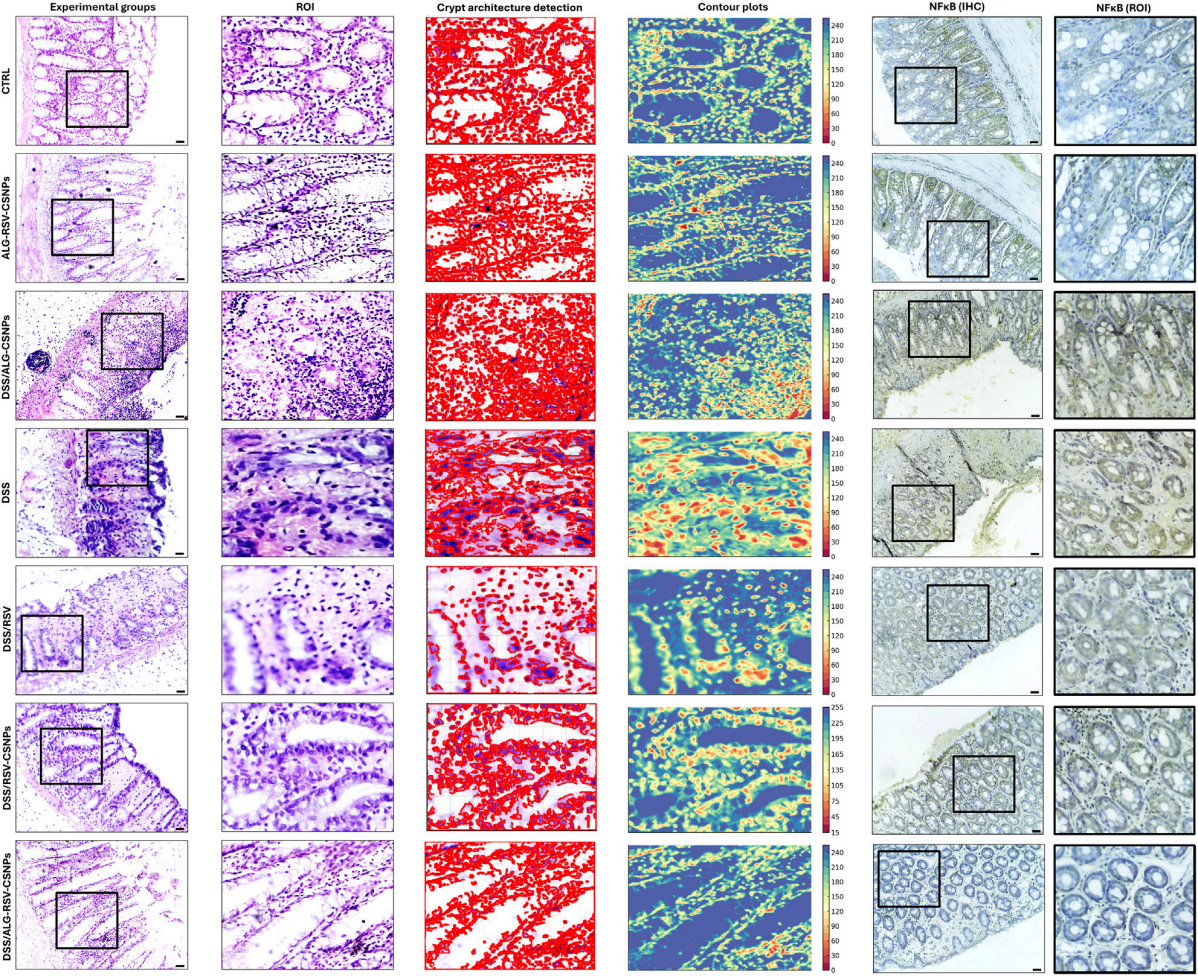
Representative microimages displaying H&E-stained sections, regions of interest (ROI), crypt architecture detection, contour plots and immunohistochemical labeling of NFκB. Column 1, histological sections; Column 2 (ROI), magnified regions of interest focusing on crypt structures; Column 3 (crypt architecture detection), crypts identified to assess crypt integrity; Column 4 (contour plots), contour plots showing structural variability with color-coded regions to differentiate damage or abnormality. The DSS and DSS/ALG-CSNPs groups show the most crypt distortion whereas groups treated with RSV and RSV-CSNPs show partial restoration of crypt structure. In contrast, ALG-RSV-CSNPs ameliorated DSS-induced distortion in the tissue architecture. Column 5 (NFκB IHC) and column 6 displays magnified regions of interest focusing on crypt structures stained with NFκB IHC. Scale bar = 50 μm. n = 6. ALG, Alginate; ALG-RSV-CSNPs, Alginate-coated rosuvastatin-loaded chitosan nanoparticles; CSNPs, Chitosan nanoparticles; CTRL, Untreated healthy control rats; DSS, Dextran sulfate sodium; DSS/ALG-CSNPs, DSS-induced rats treated with alginate-coated chitosan nanoparticles; DSS/ALG-RSV-CSNPs, DSS-induced rats treated with alginate-coated rosuvastatin-loaded chitosan nanoparticles; DSS/RSV, DSS-induced rats treated with plain rosuvastatin; DSS/RSV-CSNPs, DSS-induced rats treated with rosuvastatin-loaded chitosan nanoparticles; IHC, Immunohistochemistry; NFκB, Nuclear factor kappa-B; NFκB (IHC), Immunohistochemical staining for NFκB expression; NFκB (ROI), Magnified region of interest showing NFκB immunostaining; ROI, Region of interest; RSV, Rosuvastatin.

Figure 6 illustrates the histological findings across experimental groups. Regions of interest were analyzed for crypt structure, tissue integrity, and inflammatory cell infiltration. In the CTRL group, colonic sections revealed well-organized mucosal layers with intact crypt architecture, no crypt loss, and minimal inflammatory infiltration. The corresponding crypt architecture detection plot and contour plot showed uniform structure with minimal variability, supporting the observed tissue stability. The ALG-RSV-CSNPs group, which did not undergo DSS induction, displayed comparable histology to the CTRL group. Crypt morphology appeared intact, and inflammation was absent. This indicates that the formulation did not induce any histological changes or tissue disruption. The DSS group exhibited extensive colonic damage, including marked crypt distortion, loss of crypt density, and widespread inflammatory infiltration. Crypts were shortened or absent in some areas, and epithelial erosion was evident. The contour plots showed highly irregular topography, confirming significant structural deterioration consistent with active colitis. Crypt architecture detection plots displayed damaged crypts. Similarly, the DSS/ALG-CSNPs group displayed substantial crypt destruction and mucosal damage, comparable to the DSS group. Inflammatory infiltration remained prominent, suggesting limited therapeutic effect from the unloaded ALG-CSNPs. In contrast, DSS/RSV treatment led to partial restoration of mucosal architecture. While some areas still exhibited damage, crypts appeared more continuous, and inflammation was reduced. Crypt architecture detection plots and contour plots showed moderate reduction in structural variability. The DSS/RSV-CSNPs group showed further improvement. Crypt density was higher, and mucosal restoration was more apparent than with RSV alone. Inflammatory cell presence was visibly lower, and crypt architecture detection plots and contour plots reflected more consistent architecture. The DSS/ALG-RSV-CSNPs group demonstrated the most preserved architecture among all DSS-treated groups. Crypts were nearly restored to normal shape and distribution, and inflammatory infiltration was minimal. Crypt architecture detection plots and contour plots confirmed the highest degree of tissue recovery with minimal variability. NFκB p65 immunohistochemistry provided additional insight into inflammation. Low p65 expression was observed in CTRL and ALG-RSV-CSNPs groups. In contrast, DSS and DSS/ALG-CSNPs showed strong nuclear p65 staining, indicating active NFκB signaling and inflammation. DSS/RSV and DSS/RSV-CSNPs showed reduced expression. The DSS/ALG-RSV-CSNPs group exhibited the lowest p65 expression among DSS-treated rats, supporting its anti-inflammatory effect.

#### 3.6.3 ALG-RSV-CSNPs reduced edema, crypt atrophy, and inflammation scores in DSS-induced colitis

**FIGURE 7:**
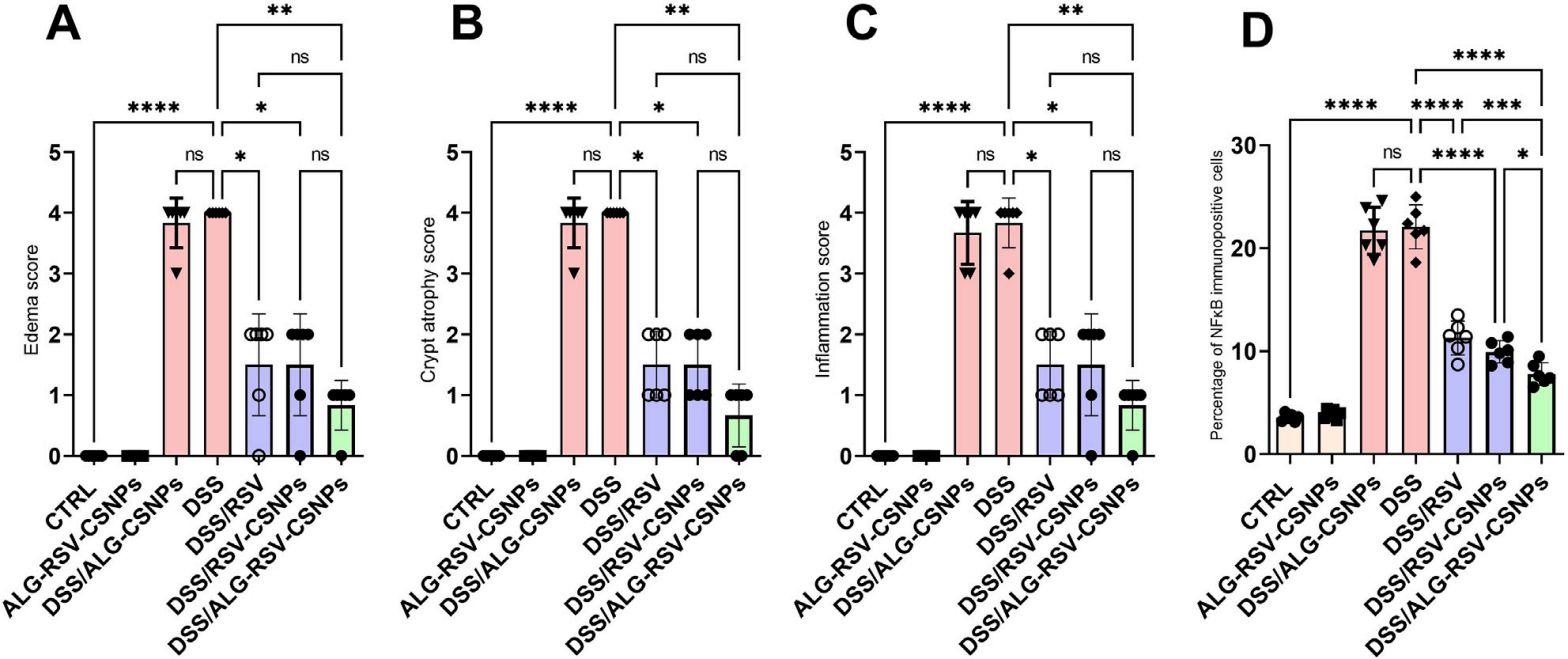
Bar graphs show results of edema score **(A)**, crypt atrophy score **(B)**, inflammation score **(C)**, and percentage NF_k_B immunopositive cells for different treatment groups. Data are expressed as median ± IQR. Statistical analysis was performed using the Kruskal–Wallis test, followed by Dunn’s *post hoc* test for multiple comparisons. n = 6. *, p < 0.05; **, p < 0.01; ***, p < 0.001; and ****, p < 0.0001. ALG, alginate; ALG-RSV-CSNPs, Alginate-coated rosuvastatin-loaded chitosan nanoparticles; CSNPs, Chitosan nanoparticles; CTRL, Control (untreated healthy rats); DSS, Dextran sulfate sodium; DSS/ALG-CSNPs, DSS-induced rats treated with alginate-coated chitosan nanoparticles; DSS/ALG-RSV-CSNPs, DSS-induced rats treated with alginate-coated rosuvastatin-loaded chitosan nanoparticles; DSS/RSV, DSS-induced rats treated with plain rosuvastatin; DSS/RSV-CSNPs, DSS-induced rats treated with rosuvastatin-loaded chitosan nanoparticles; NFκB, Nuclear factor kappa-B; RSV, Rosuvastatin.

As depicted in Figure 7, the CTRL and ALG-RSV-CSNPs groups exhibited the lowest scores, indicating no edema, crypt atrophy, or inflammation. The DSS group showed the highest scores in all three parameters. Notably, the DSS/ALG-RSV-CSNPs group exhibited significant reductions in these scores compared to the DSS group, which showed the greatest therapeutic efficacy in reducing colitis-associated damage. The DSS/RSV and DSS/RSV-CSNPs groups exhibited less pronounced effects than in the DSS/ALG-RSV-CSNPs group. The DSS/ALG-CSNPs and DSS groups showed significant increase in the percentage NFκB immunopositive cells compared to the CTRL group. However, significant reductions were found in the DSS/RSV, DSS/RSV-CSNPs and in particular in the DSS/ALG-RSV-CSNPs group in contrast to the DSS group.

#### 3.6.4 ALG-RSV-CSNPs exhibited enhanced antioxidant effects in DSS-induced colitis

**FIGURE 8:**
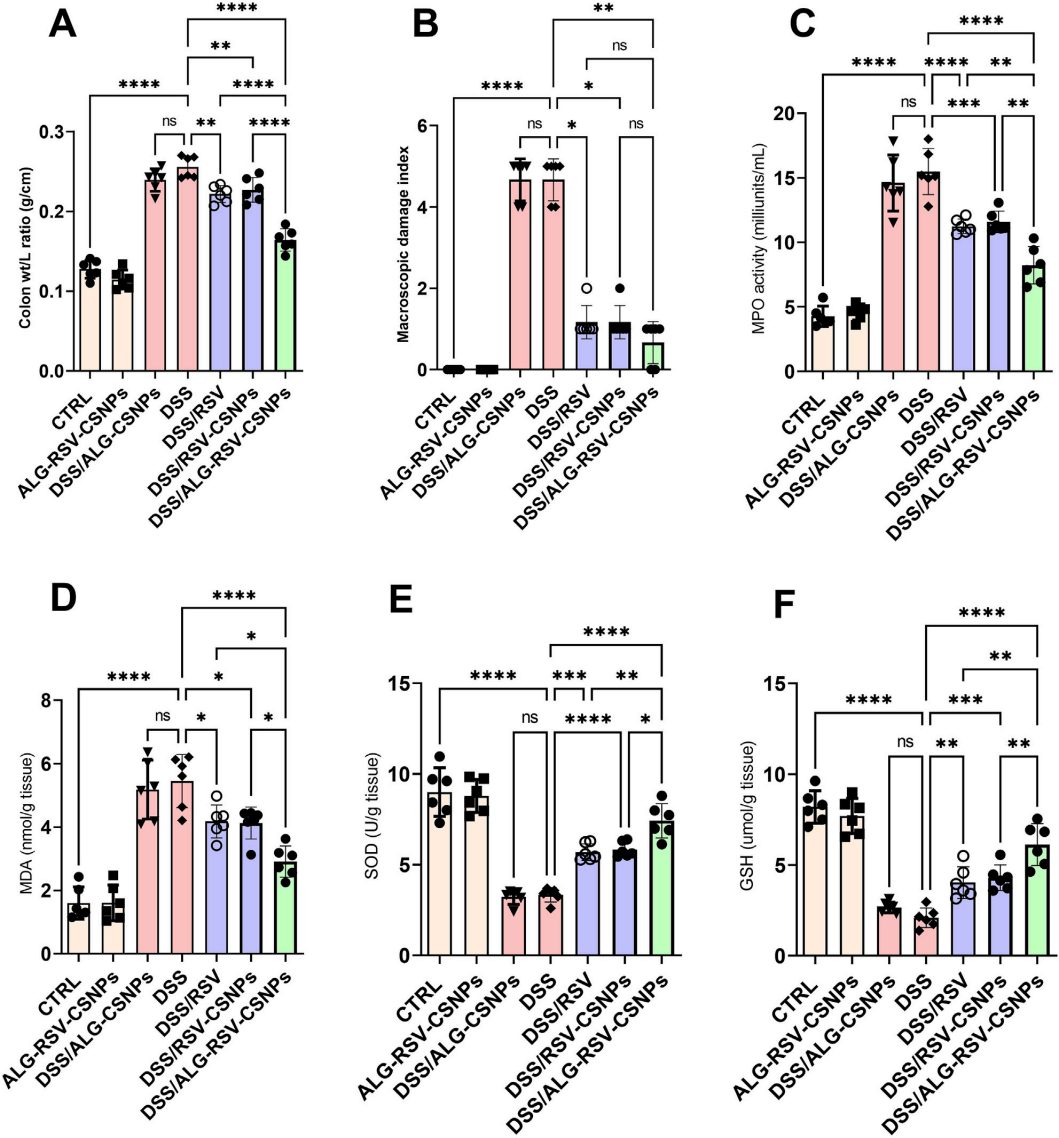
Assessment of colon weight-to-length ratio **(A)** MDI **(B)** MPO activity **(C)** MDA levels **(D)** SOD activity **(E)** and GSH levels **(F)** in DSS-induced colitis model. Data are expressed as mean ± SD. Statistical analysis was performed using one-way ANOVA, followed by Tukey’s *post hoc* test for multiple comparisons. n = 6. *, p < 0.05; **, p < 0.01; ***, p < 0.001; and ****, p < 0.0001. ALG, Alginate; ALG-RSV-CSNPs, Alginate-coated rosuvastatin-loaded chitosan nanoparticles; CSNPs, Chitosan nanoparticles; CTRL, Control (untreated healthy rats); DSS, Dextran sulfate sodium; DSS/ALG-CSNPs, DSS-induced rats treated with alginate-coated chitosan nanoparticles; DSS/ALG-RSV-CSNPs, DSS-induced rats treated with alginate-coated rosuvastatin-loaded chitosan nanoparticles; DSS/RSV, DSS-induced rats treated with plain rosuvastatin; DSS/RSV-CSNPs, DSS-induced rats treated with rosuvastatin-loaded chitosan nanoparticles; GSH, Reduced glutathione; MDA, Malondialdehyde; MPO, Myeloperoxidase; RSV, Rosuvastatin; SOD, Superoxide dismutase.

As depicted in Figure 8, the DSS group exhibited a significant increase in both the colon weight-to-length ratio and the macroscopic damage index. Both DSS/RSV and DSS/RSV-CSNPs treatments significantly reduced these measures, but the reductions were more pronounced in the DSS/ALG-RSV-CSNPs group. Additionally, MPO activity and MDA levels were significantly elevated in the DSS group. Again, treatment with DSS/RSV and DSS/RSV-CSNPs resulted in moderate reductions in both MPO and MDA but the DSS/ALG-RSV-CSNPs group exhibited the most significant reductions in both parameters. Furthermore, the DSS group showed a marked reduction in both SOD activity and GSH levels. While DSS/RSV and DSS/RSV-CSNPs significantly improved both SOD activity and GSH levels, the DSS/ALG-RSV-CSNPs group showed the greatest restoration of these antioxidant markers. Collectively, these findings suggest that ALG-RSV-CSNPs more effectively enhance the colonic antioxidant defense system showing the most robust anti-inflammatory effect reducing tissue damage in DSS-induced colitis.

#### 3.6.5 ALG-RSV-CSNPs exhibited superior anti-inflammatory and anti-apoptotic effects in DSS-induced colitis

**FIGURE 9:**
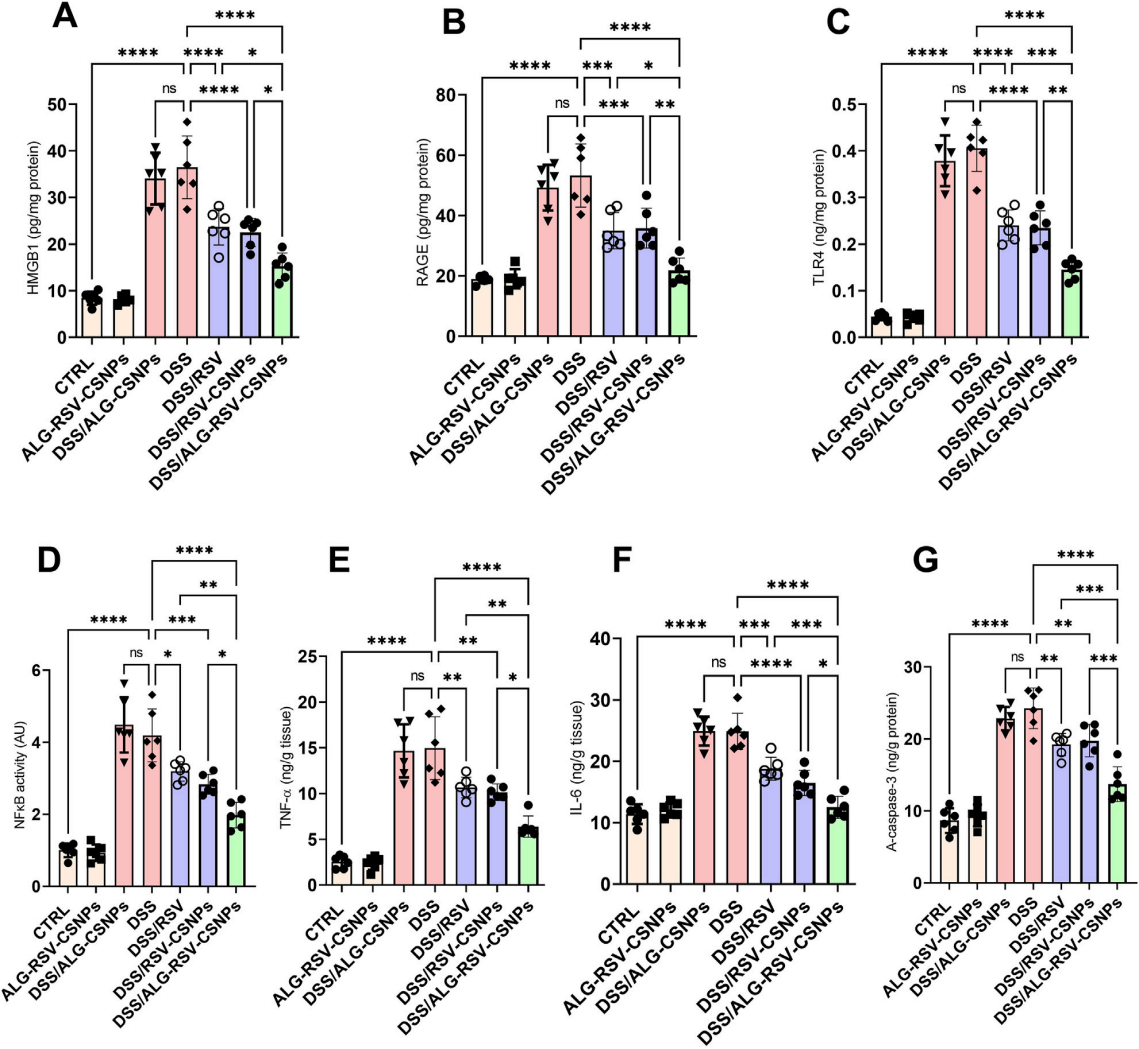
**(A)** HMGB1 levels. **(B)** RAGE levels. **(C)** TLR4 expression. **(D)** NFκB activity. **(E)** TNF-α levels. **(F)** IL-6 levels. **(G)** Active caspase-3 levels. Data are expressed as mean ± SD. Statistical analysis was performed using one-way ANOVA and Tukey’s *post hoc* test for multiple comparisons. n = 6. *, p < 0.05; **, p < 0.01; ***, p < 0.001; and ****, p < 0.0001. ALG, Alginate; ALG-RSV-CSNPs, Alginate-coated rosuvastatin-loaded chitosan nanoparticles; AU, Arbitrary units; CSNPs, Chitosan nanoparticles; CTRL, Control (untreated healthy rats); DSS, Dextran sulfate sodium; DSS/ALG-CSNPs, DSS-induced rats treated with alginate-coated chitosan nanoparticles; DSS/ALG-RSV-CSNPs, DSS-induced rats treated with alginate-coated rosuvastatin-loaded chitosan nanoparticles; DSS/RSV, DSS-induced rats treated with plain rosuvastatin; DSS/RSV-CSNPs, DSS-induced rats treated with rosuvastatin-loaded chitosan nanoparticles; HMGB1, High mobility group box 1; IL-6, Interleukin-6; NFκB, Nuclear factor kappa-B; RAGE, Receptor for advanced glycation end products; RSV, Rosuvastatin; TLR4, Toll-like receptor 4; TNF-α, Tumor necrosis factor alpha; A-caspase-3, Active caspase-3.

As depicted in Figure 9, HMGB1 and RAGE levels were significantly elevated in the DSS group. Both DSS/RSV and DSS/RSV-CSNPs treatments reduced these levels. However, DSS/ALG-RSV-CSNPs showed the greatest reduction. Additionally, TLR4 expression and NFκB activity were significantly higher in the DSS group. While both DSS/RSV and DSS/RSV-CSNPs treatments significantly lowered these levels, the DSS/ALG-RSV-CSNPs group exhibited the most substantial reduction in both markers. Moreover, TNF-α and IL-6 levels were significantly elevated in the DSS group. Both DSS/RSV and DSS/RSV-CSNPs treatments led to significant reductions. However, DSS/ALG-RSV-CSNPs showed the most pronounced decrease in both cytokines. Furthermore, the DSS group exhibited high levels of active caspase-3. While both DSS/RSV and DSS/RSV-CSNPs treatments reduced caspase-3 levels, the DSS/ALG-RSV-CSNPs treatment group showed the most substantial decrease. These findings indicate superior anti-inflammatory and anti-apoptotic effects of ALG-RSV-CSNPs over plain RSV and RSV-CSNPs.

#### 3.6.6 Strong positive correlations and nonlinear influences of RAGE and TLR4 on NFκB activity in DSS-induced colitis

**FIGURE 10:**
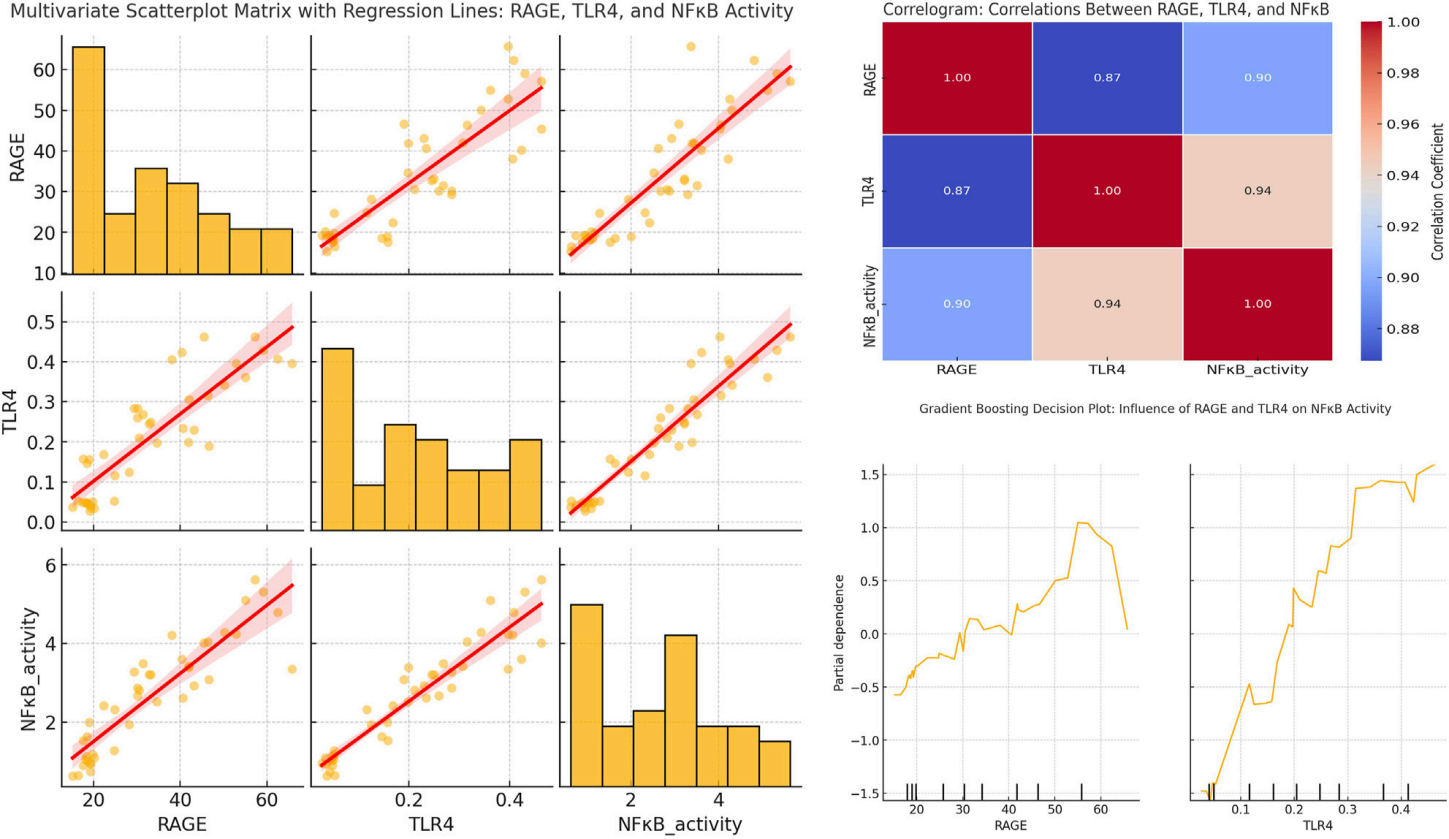
Multivariate scatterplot matrix with regression lines, correlogram, and gradient boosting partial dependence plots showing the relationships between RAGE, TLR4, and NFκB activity. The scatterplot matrix (left) includes histograms and regression lines for each pair of variables. The correlogram (top right) displays Pearson correlation coefficients. The Gradient Boosting PDPs (bottom right) illustrate the marginal effects of RAGE and TLR4 on NFκB activity. NFκB, Nuclear factor kappa-B; NFκB activity, NFκB transcriptional activity; RAGE, Receptor for advanced glycation end products; TLR4, Toll-like receptor 4.

As shown in Figure 10, the scatter plot matrix demonstrates strong positive relationships between RAGE, TLR4, and NFκB activity across all experimental groups. Notably, both RAGE and TLR4 significantly influenced NFκB activation. The plot between RAGE and NFκB shows a clear positive linear trend, indicating that as RAGE expression escalates, NFκB activity also rises (correlation coefficient: 0.90). Notably, the relationship between TLR4 and NFκB was even stronger. It demonstrated a steeper upward trend and a higher correlation coefficient (0.94). This relationship suggests that TLR4 has a more direct and potent effect on NFκB activation. These findings advocate that both receptors are important in driving NFκB-mediated inflammatory response. Besides, TLR4 may play a more dominant role in enhancing the inflammation state across various experimental groups, including those treated with DSS, RSV, RSV-CSNPs, and ALG-RSV-CSNPs.

The ALG-RSV-CSNPs group exhibited the most significant reduction in RAGE, TLR4, and NFκB activity. This result highlights the superior efficacy of this treatment compared to plain RSV and RSV-CSNPs. Both RSV-CSNPs and plain RSV also showed moderate reductions in these markers. However, the effect was less pronounced than in the ALG-RSV-CSNPs group. In contrast, the control DSS group displayed the highest levels of RAGE, TLR4, and NFκB activity.

The correlogram visually reinforces the strong positive relationships among these variables across all experimental groups. The correlation between RAGE and NFκB activity remains strong (coefficient: 0.90) in the DSS group and is gradually attenuated in the RSV, RSV-CSNPs, and ALG-RSV-CSNPs groups. Notably, the most significant reduction observed in the ALG-RSV-CSNPs treatment group. Similarly, the strongest correlation between TLR4 and NFκB activity (coefficient: 0.94) is present in the DSS group and is progressively reduced in the treatment groups, particularly under ALG-RSV-CSNPs treatment. These findings emphasize its role in dampening RAGE- and TLR4-driven NFκB activation.

The gradient boosting partial dependence plots (PDPs) provide additional insights into the nonlinear effects of RAGE and TLR4 on NFκB activity across the treatment groups. The PDP for RAGE shows that NFκB activity remains stable at lower RAGE levels. However, the NFκB activity rises sharply after a threshold of 35–40 units to peak between 55 and 60 units of RAGE expression. This nonlinear relationship is most pronounced in the DSS group, with the ALG-RSV-CSNPs treatment effectively shifting this threshold, resulting in reduced NFκB activation. On the other hand, the PDP for TLR4 shows a more linear and consistent increase in NFκB activity as TLR4 expression rises. After reaching a threshold of approximately 0.25–0.30 units of TLR4 expression, there is a steeper rise in NFκB activity. In view of this, the ALG-RSV-CSNPs group showed the greatest reduction in this effect indicating its role in mitigating TLR4-driven inflammation after this threshold is surpassed.

#### 3.6.7 Predictive modeling highlights HMGB1, RAGE, TLR4, and NFκB as key determinants of colitis severity mitigated by ALG-RSV-CSNPs

**FIGURE 11:**
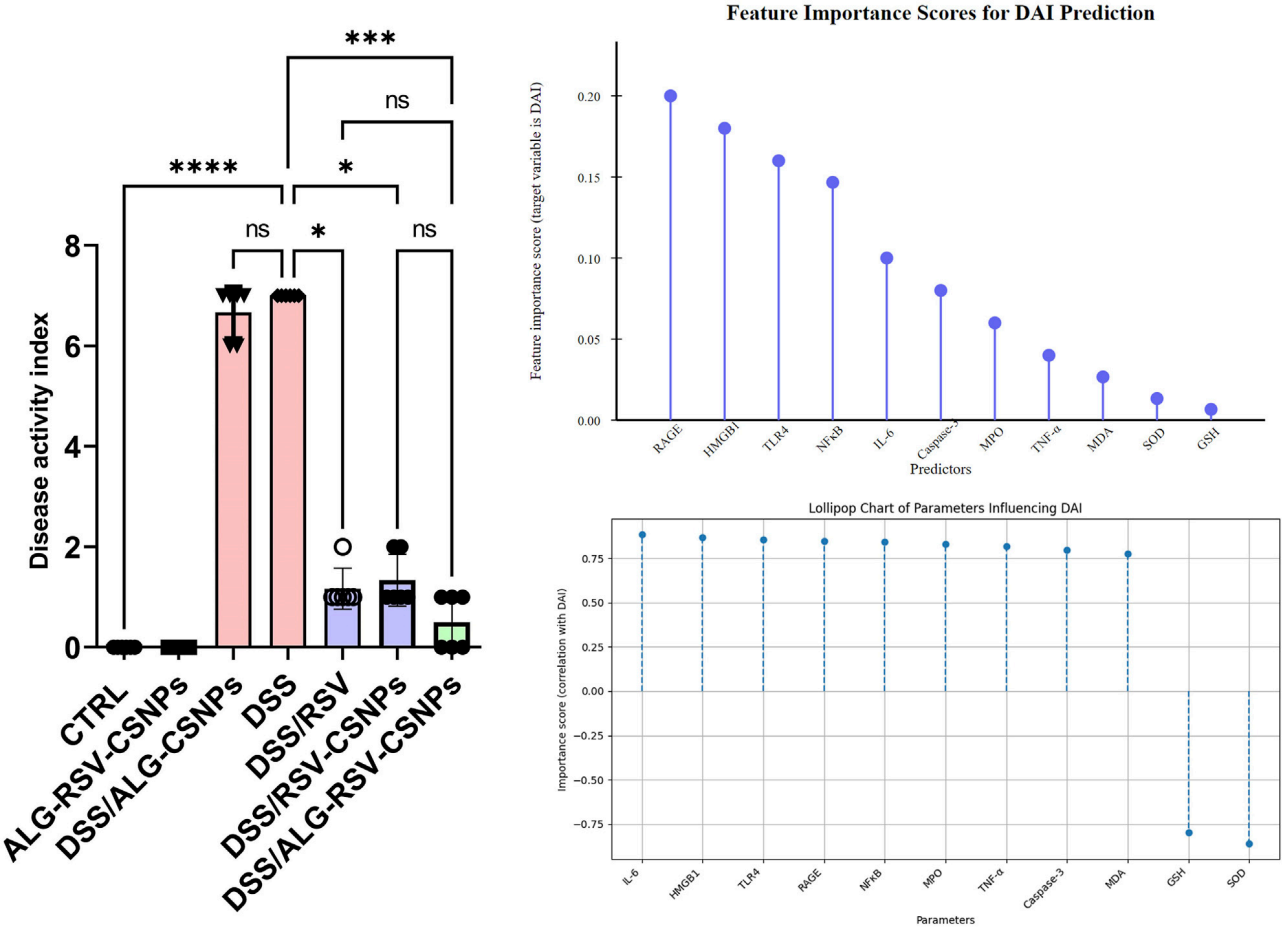
Assessment of DAI and the importance of biochemical markers in predicting DAI. The left panel displays the DAI for various experimental groups, while the top right lollipop chart illustrates the feature importance scores of different markers for DAI prediction. The bottom right chart shows their correlation with DAI. In the bar graph, data are expressed as median ± IQR. Statistical analysis was performed using the Kruskal–Wallis test, followed by Dunn’s *post hoc* test for multiple comparisons. *, p < 0.05; **, p < 0.01; ***, p < 0.001; and ****, p < 0.0001. ALG, Alginate; ALG-RSV-CSNPs, Alginate-coated rosuvastatin-loaded chitosan nanoparticles; Caspase-3, Active caspase-3; CSNPs, Chitosan nanoparticles; CTRL, Control (untreated healthy rats); DAI, Disease activity index; DSS, Dextran sulfate sodium; DSS/ALG-CSNPs, DSS-induced rats treated with alginate-coated chitosan nanoparticles; DSS/ALG-RSV-CSNPs, DSS-induced rats treated with alginate-coated rosuvastatin-loaded chitosan nanoparticles; DSS/RSV, DSS-induced rats treated with plain rosuvastatin; DSS/RSV-CSNPs, DSS-induced rats treated with rosuvastatin-loaded chitosan nanoparticles; GSH, Reduced glutathione; HMGB1, High mobility group box 1; IL-6, Interleukin-6; MDA, Malondialdehyde; MPO, Myeloperoxidase; NFκB, Nuclear factor kappa-B; RAGE, Receptor for advanced glycation end products; RSV, Rosuvastatin; SOD, Superoxide dismutase; TNF-α, Tumor necrosis factor alpha.

The bar graph in Figure 11 presents the disease activity (measured as the DAI) across various experimental groups. The DSS/ALG-RSV-CSNPs group demonstrated the most significant reduction in DAI compared to the DSS group. On the other hand, DSS/RSV-CSNPs and DSS/RSV treatments showed moderate reductions in DAI, suggesting some improvement. However, the effect is not as profound as seen with the combined ALG-RSV-CSNPs treatment. In the feature importance analysis (top right panel), the predictor RAGE, HMGB1, TLR4, and NFκB emerged as the most critical variable influencing DAI, with the highest feature importance score (∼0.10–0.20). This underscores their dominant role in predicting DAI, particularly in the DSS group, where inflammation is at its peak. Further analysis using correlation coefficients (bottom right panel) reveals that markers such as IL-6, HMGB1, TLR4, RAGE, and NFκB exhibit moderate positive correlations with DAI. These findings indicate that higher levels of these inflammatory markers are linked to greater disease severity, particularly in the DSS group where inflammation is most pronounced. Collectively, these results suggest that HMGB1, RAGE, TLR4, and NFκB are key contributors to increased DAI.

## 4 Discussion

Oral colonic drug delivery is preferable over systemic or rectal routes in the case of chronic diseases such as UC. However, achieving effective delivery to the colon presents physiological challenges, as the drug is required to be passed through the GIT, which is characterized by varying enzymatic activity and pH levels that can negatively affect drug delivery to the colon (Zhang et al., 2017). To address this, a pH-dependent delivery system was fabricated, consisting of RSV-CSNPs as the core, and ALG as a coat.

The rationale beyond this selection is that ALG and CS are renowned for their favorable biocompatibility, biodegradability, and low toxicity as natural polymers (Kulig et al., 2016; Kothale et al., 2020). Also, the cationic nature of CSNPs can electrostatically interact with anionic ALG under simple, mild conditions without the need for organic solvents, facilitating the formation of a stable delivery system (Borges et al., 2005). The complementary properties of ALG’s acidic stability and CS’s resistance to alkaline environments offer synergistic benefits that can navigate the complexities of GIT while delivering RSV to its target location in the colon, enhancing its therapeutic efficacy against UC.

Being an acidic stable polymer, ALG can overcome the limitations of CSNPs’ degradation in an acidic microenvironment. It can also protect CSNPs from the harsh acidic and enzymatic microenvironment within the stomach until they reach intact within the small intestine (pH 7.4). At this stage, the exposed CSNPs after the degradation of the ALG coat can decrease the premature release of RSV owing to the limited swelling ability at this pH. In the colonic environment (pH 6.8), the CSNPs facilitated prolonged delivery of RSV that is consistent with the colon’s prolonged transit time (60–72 h) (Amidon et al., 2015), thus improving the availability and therapeutic concentration of the drug in the colon.

Moreover, the significance of ALG coating can be expanded on its ability to enhance nanoparticle stability through both steric and electrostatic mechanisms. High grafting densities of ALG on nanoparticle surfaces can lead to robust steric stabilization, reducing aggregation tendencies. This is supported by studies (Wen et al., 2023), demonstrating that NPs with dense polymer brushes exhibit improved colloidal stability in various media. Additionally, the electrostatic repulsion between charged ALG coatings contributes to maintaining nanoparticle dispersion stability.

Recent studies have highlighted the advantages of chitosan-alginate nanoparticle systems in drug delivery, emphasizing their biocompatibility, biodegradability, and ability to offer controlled release in the GIT by providing a pH-sensitive release profile and enhanced stability against gastric degradation (Li et al., 2022; Audrey Masindi, 2024; Zabihollahi et al., 2024). These systems have been explored for their potential in targeted delivery, especially in the treatment of diseases like colon cancer and UC, where the ability to deliver drugs directly to the colon is critical. Our formulation, which integrates RSV-ChNPs coated with ALG, builds on these systems, focusing on integration of factorial design and response optimization analysis, with experimental validation which provides a comprehensive understanding of how various process variables affect formulation properties. This approach enhances the precision of formulation optimization, contributing to the novelty and effectiveness of our colonic drug delivery system.

Initially, five different formulations of CSNPs were prepared after crosslinking with Na-TPP under mild conditions. These formulations were characterized to determine the optimal formulation for the ALG-coating process. Changing the mass ratios of CS and Na-TPP from 2:1 to 6:1 had a significant impact on the evaluated characteristics. The prepared formulations showed uniform size distribution, a positive surface charge, and a relatively high EE%. The favorable characteristics of formulation F3, such as the smallest PS, PDI, and highest EE%, position it as an optimal candidate for further development.

Once the optimal CSNPs formulation was chosen, the following step was the ALG-coating procedure. The effective completion of this procedure relies on the interaction between cationic CSNPs and anionic ALG polymers, achieved by mild agitation (Fan et al., 2006). However, a significant challenge during the coating process was DL, which could negatively affect the amount of RSV entrapped in CSNPs. To address this challenge, the effect of different variables, such as, ALG concentrations, stirring time, and tween 80 incorporation were evaluated *via* a 2^3^ full factorial design, aiming to minimize DL%. In addition to achieving a low DL%, the design also investigated the influence of variables to achieve a low PS, Q_2_%, and Q_6_%.

The selection of 3% w/v as the lower limit for the ALG concentration was based on preliminary trials, where concentrations lower than this threshold resulted in precipitation within the reaction medium. This behavior was also observed in previous studies (Borges et al., 2005; Borges et al., 2006; Li et al., 2008). The precipitation likely occurred due to an insufficient reduction in the positive charge of CSNPs at lower concentrations, leading to particle aggregation. At 3% and 4%, the ZP significantly increased (ranging from −25.9 mV to −36.4 mV), ensuring stability without aggregation. The upper concentration limit of 4% was chosen as using higher ALG concentrations (5%) led to a slight increase in the viscosity of the coating medium, making proper mixing more difficult. Furthermore, when using 5% ALG, the measured PS increased to 737.5 nm with a higher PDI of 0.9. Therefore, we selected 3% and 4% ALG concentrations, as they ensure better stability in the formulation, allowing for effective electrostatic interactions between the cationic CSNPs and anionic ALG, resulting in stable and well-coated nanoparticle formulations. According to the factorial design, eight different formulations of ALG-coated CSNPs (Fr1–Fr8) were prepared. The increase in PS and the conversion of the surface charge to negative values revealed the presence of the ALG layer around CSNPs, which was further confirmed by TEM and FTIR studies.

The competitive electrostatic interaction between the anionic ALG polymer and the negatively charged carboxylate groups of RSV during their interaction with cationic CSNPs accounts for the DL that occurs during the coating process. Consequently, increasing ALG concentration and stirring time significantly increase this competition, resulting in high DL%. However, incorporating tween 80 into the reaction medium reduces competition, resulting in a low DL%. Tween 80 could function as a steric stabilizer (Scolari et al., 2019), potentially disrupting the electrostatic interaction between ALG and CS. Moreover, it may potentially form a coating on the CSNPs, therefore causing steric hindrance with the RSV loss from CSNPs.

Regarding the drug release patterns, the objective was to achieve sustained drug release within the colon without the risk of excessive premature release in the stomach or small intestine. As a result, our goal was to get a possible minimum value of Q_2_% and Q_6_%. The protective role of ALG-coating was confirmed by the lower release percentage observed after 2 h at gastric pH. Increasing the stirring time and ALG concentration, resulted in a thicker layer of ALG, which is acidic stable polymer, decreasing the Q_2_%. Moreover, the presence of tween 80 as an additional coat further decreased Q_2_%. At intestinal pH (7.4), as expected, nor ALG concentration neither stirring time affected Q_6_%. However, interestingly, the addition of tween 80 significantly decreased Q_6_%. This may be explained by the steric stabilizer effect of tween 80 around CSNPs, decreasing drug release.

The formulation (Fr2) was the best among the different ALG-coated CSNP formulations, with a DL% of 0.88% ± 0.09%, a Q_2_% of 6.812% ± 1.06%, and a Q_6_% of 25.41% ± 2.03%. The lower release% associated with the optimized formulation compared to the uncoated CSNPs underscores the potential role of ALG coating and tween 80 addition in the development of an effective colonic delivery system that can control premature release within the stomach and the intestine and sustain RSV release within the colon, enhancing drug concentration for a long time within the colonic microenvironment.

The animal study highlights the superior therapeutic potential of ALG-RSV-CSNPs in mitigating DSS-induced colitis, particularly when compared to plain RSV and RSV-CSNPs groups. Across various inflammatory and oxidative stress markers, ALG-RSV-CSNPs consistently demonstrated more pronounced reductions, suggesting an enhanced mechanism of action. This is likely due to the targeted delivery and sustained release of RSV in the colonic environment, allowing the drug to stay at the inflammatory site for a long time.

Histopathological evaluations revealed that ALG-RSV-CSNPs markedly reduced inflammation, and decreased edema scores compared to RSV and RSV-CSNPs groups. Additionally, although both RSV-CSNPs and plain RSV formulations showed improvements in crypt architecture, the effects were less pronounced compared to ALG-RSV-CSNPs. These improvements highlight that the combination of ALG and CS polymers plays a crucial role in preventing premature drug degradation or release in the upper GIT, ensuring more of the RSV reaches the inflamed colon, thereby providing enhanced mucosal protection and more efficiently repairing intestinal epithelial damage.

From a mechanistic standpoint, the superior efficacy of ALG-RSV-CSNPs can be attributed to their enhanced ability to modulate oxidative stress and inflammation. Oxidative stress markers such as MPO and MDA, which were significantly elevated in the DSS group, were effectively reduced following treatment with ALG-RSV-CSNPs. This reduction was more significant compared to the RSV and RSV-CSNPs groups, suggesting that the coated formulation allows for better scavenging of ROS and protection against lipid peroxidation. The enhanced activity of SOD and GSH in the ALG-RSV-CSNPs group further supports the notion that this formulation provides robust antioxidant defense, which is critical in alleviating DSS-induced oxidative damage (Cavalu et al., 2022). Although RSV-CSNPs and plain RSV showed improvements in these parameters, the ALG-RSV-CSNPs group consistently exhibited stronger antioxidant effects, likely due to the localized release of RSV in the colon, which ensures a prolonged therapeutic effect on the site of oxidative stress. Therefore, the antioxidant effects appear to be both RSV-specific and formulation-enhanced. While treatment with plain RSV led to moderate improvements in SOD activity, GSH levels, and MDA reduction, consistent with its known antioxidant properties, the effects were more pronounced with RSV-CSNPs and maximized with ALG-RSV-CSNPs. This pattern suggests that RSV itself contributes to the modulation of oxidative stress pathways, but the nanoparticle-based delivery system, particularly the alginate coating, enhances these effects by improving colonic targeting, bioavailability, and sustained release. Therefore, the antioxidant activity reflects both the intrinsic pharmacological action of RSV and the added benefit of nanoparticle-mediated delivery.

The impact of ALG-RSV-CSNPs on inflammatory markers was also significantly greater compared to RSV and RSV-CSNPs. NFκB activity, a key regulator of inflammation (Liu et al., 2017; Abdelhamid et al., 2022), was most effectively suppressed in the ALG-RSV-CSNPs group, which corresponds with the more substantial reductions in pro-inflammatory cytokines like TNF-α, IL-6, and HMGB1 (Lin et al., 2018; Hiramoto et al., 2020; Chen R. et al., 2022). This potent anti-inflammatory action is likely due to the enhanced targeting of the ALG-RSV-CSNPs to inflamed tissues, allowing for better suppression of inflammatory signaling pathways. While RSV-CSNPs and plain RSV also led to reductions in these cytokines, their effects were less pronounced, indicating the improved colonic delivery associated with the coated formulation, which in turn enhanced the anti-inflammatory response.

Additionally, the scatter plot analysis shows that both RAGE and TLR4 are strongly correlated with NFκB activation, which drives inflammatory responses. The data suggests that the ALG-RSV-CSNPs formulation is particularly effective in reducing these correlations, thereby preventing the amplification of inflammation. By reducing RAGE and TLR4 expression, ALG-RSV-CSNPs interrupt the feed-forward loop of inflammation mediated by NFκB, which is likely a key mechanism underlying its superior therapeutic effects. Although RSV-CSNPs and plain RSV also modulated these pathways, ALG-RSV-CSNPs achieved a more substantial reduction in NFκB activity, indicating that the formulation may be better suited for disrupting inflammatory signaling at multiple levels.

Our results and those of others emphasize the importance of oxidative stress and inflammation in driving disease activity which was assessed as DAI (Saber et al., 2021b; Abdelhamid et al., 2022; Tratenšek et al., 2024). The findings shown in lollipop charts demonstrate that MPO, MDA, SOD, and GSH play critical roles in determining disease severity, with ALG-RSV-CSNPs exerting the most profound effects on these markers. The feature importance and correlation coefficient analysis show that while both RSV-CSNPs and plain RSV had moderate effects on these key markers, ALG-RSV-CSNPs showed the most substantial modulation of DAI, further confirming its superiority.

The findings from this study strongly suggest that the ALG-RSV-CSNPs formulation exerts its superior anti-inflammatory effects through effective inhibition of the HMGB1-driven RAGE and TLR4 signaling through NFκB. HMGB1-activated RAGE/TLR4-NFκB signaling is a key regulator of inflammatory responses in DSS-induced colitis (Reed et al., 2016; Chen et al., 2020; Yang et al., 2020). HMGB1, a nuclear protein released during cellular stress and injury, acts as a damage-associated molecular pattern (DAMP) that binds to RAGE and activates downstream inflammatory pathways, notably NFκB (Wang et al., 2019; Chen R. et al., 2022). The significant reductions in HMGB1 and RAGE levels observed in the ALG-RSV-CSNPs group indicate that this treatment effectively disrupts the initial activation of the HMGB1/RAGE axis, thereby limiting the propagation of the inflammatory cascade. By inhibiting RAGE expression, ALG-RSV-CSNPs reduce the receptor’s ability to bind HMGB1 and other ligands, preventing the sustained activation of NFκB and the release of pro-inflammatory cytokines such as TNF-α and IL-6. While RSV-CSNPs and plain RSV also led to reductions in HMGB1 and RAGE, the effects were less pronounced, suggesting that the enhanced delivery of RSV in the ALG-RSV-CSNPs formulation allows for more effective targeting of this pathway.

The TLR4/NFκB pathway also plays a crucial role in colitis pathogenesis and interacts with the HMGB1/RAGE axis to further amplify the inflammatory response (Wei et al., 2022). TLR4 is a pattern recognition receptor (PRR) that detects pathogen-associated molecular patterns (PAMPs) and DAMPs (Kim et al., 2023), including HMGB1 (He et al., 2018), leading to the activation of downstream signaling cascades, particularly NFκB (Gargiulo et al., 2015; Saber et al., 2022). The strong positive correlation between TLR4 expression and NFκB activity observed in the scatter plot analysis highlights the role of TLR4 in driving NFκB-mediated inflammation. ALG-RSV-CSNPs were particularly effective in downregulating TLR4 expression, thus limiting NFκB activation through this secondary pathway. By targeting both RAGE and TLR4, ALG-RSV-CSNPs effectively interrupt two major routes of NFκB activation, resulting in a more comprehensive suppression of inflammatory responses compared to RSV-CSNPs and plain RSV. This dual inhibition of TLR4 and RAGE, both of which contribute to NFκB activation, underscores the mechanistic superiority of the ALG-RSV-CSNPs formulation in controlling inflammation at multiple levels, leading to improved therapeutic outcomes in DSS-induced colitis.

Overall, the enhanced *in vivo* efficacy observed with the ALG-coated RSV-CSNPs highlights the significant potential of the formulated NPs for therapeutic applications, particularly in treating inflammatory diseases like colitis. The sustained release profile of the NPs is a key factor contributing to these promising results. By ensuring gradual and controlled drug release, the formulation achieves prolonged retention in the colon, allowing for targeted action at the site of inflammation. This sustained release not only enhances the localized therapeutic effects of RSV but also minimizes premature drug release in the stomach and small intestine due to the protective barrier by ALG coating, thereby ensuring higher drug concentrations in the colonic environment. These characteristics of the formulation play a crucial role in improving efficacy and may offer a substantial therapeutic advantage, particularly in diseases requiring localized treatment.

## 5 Conclusion

Different RSV-CSNPs formulations were prepared *via* the ionic gelation method using different ratios between CS and Na-TPP. The prepared formulations were characterized regarding PS, ZP, and EE% for selecting the optimal formulation for the ALG coating process. The *in vitro* results demonstrated that the careful manipulation of the CS/Na-TPP mass ratio can significantly impact the physicochemical properties of RSV-CSNPs. The ALG coating around CSNPs was confirmed by the increase of PS and the conversion of zeta potential into negative values, further validated by TEM and FTIR analysis. Moreover, the well-established factorial design enabled the effective preparation of ALG-coated CSNPs with satisfying physicochemical properties by optimizing the parameters in the coating process. As evidenced by the minimal DL%, Q2%, and Q6% values obtained in the optimized formulation. The *in vitro* release study at different pH levels simulating the GIT demonstrated the pH-responsive release characteristics of the optimized formulation, with a significantly lower RSV release % compared to the uncoated CSNPs, emphasizing the protective role of the ALG coat. Interestingly, we found that tween 80 not only reduces DL% during the coating process but also serves as a steric stabilizer, effectively preventing premature drug release at intestinal pH (7.4), highlighting the critical role of the optimized coating process and tween 80 incorporation.

The ALG-RSV-CSNPs formulation provides significant advantages over plain RSV and RSV-CSNPs by more effectively modulating both oxidative stress and inflammatory pathways, particularly through the inhibition of the HMGB1/RAGE/NFκB signaling axis and the TLR4/NFκB pathway. This was evidenced by enhanced histological recovery, greater reductions in inflammatory markers such as HMGB1, RAGE, TLR4, and NFκB activity, and improved DAI scores. The dual inhibition of RAGE and TLR4, which converge on NFκB activation, further highlights the mechanistic superiority of ALG-RSV-CSNPs in suppressing inflammation at multiple levels. The unique combination of alginate and chitosan in the nanoparticle formulation contributes to enhanced localization, targeted delivery, and sustained release of RSV within the colon, allowing for more efficient suppression of these critical inflammatory pathways. These findings position ALG-RSV-CSNPs as a highly promising therapeutic option for treating colitis and potentially other inflammatory conditions driven by NFκB signaling.

However, clinical trials in human patients are essential to determine the efficacy of this drug delivery system in a real-world setting. Moreover, other physiological factors, such as potential interactions with gut microbiota, were not explored. Future studies could be adapted for other therapeutic agents or conditions that require targeted colon delivery. Lastly, further molecular studies are needed to fully elucidate the mechanisms behind its superior efficacy, particularly regarding its impact on pathways beyond the HMGB1-activated RAGE/TLR4-NFκB signaling. These investigations would help refine the therapeutic approach and broaden its potential applications.

However, future research should validate these findings in multiple colitis models and across both sexes to improve translational value. Longer treatment periods and follow-up studies are needed to assess relapse prevention, and safety. Clinical trials in human patients are essential to determine effectiveness in real-world settings. Other physiological factors, including interactions with gut microbiota should be explored. The approach could also be adapted for other therapeutic agents or diseases requiring targeted colon delivery. Further molecular studies are needed to clarify the mechanisms behind its superior efficacy, especially regarding pathways beyond HMGB1-activated RAGE/TLR4-NFκB signaling. Furthermore, the precise cellular targets, such as enterocytes or resident immune cells like macrophages should be distinguished in future research. These investigations would help optimize the system and expand its potential applications.

## Data Availability

The original contributions presented in the study are included in the article/supplementary material, further inquiries can be directed to the corresponding authors.
